# Antibodies Trap Tissue Migrating Helminth Larvae and Prevent Tissue Damage by Driving IL-4Rα-Independent Alternative Differentiation of Macrophages

**DOI:** 10.1371/journal.ppat.1003771

**Published:** 2013-11-14

**Authors:** Julia Esser-von Bieren, Ilaria Mosconi, Romain Guiet, Alessandra Piersgilli, Beatrice Volpe, Fei Chen, William C. Gause, Arne Seitz, J. Sjef Verbeek, Nicola L. Harris

**Affiliations:** 1 Swiss Vaccine Research Institute and Global Health Institute, École Polytechnique Fédérale de Lausanne (EPFL), Lausanne, Switzerland; 2 Bioimaging and Optics Core Facility, École Polytechnique Fédérale de Lausanne (EPFL), Lausanne, Switzerland; 3 Institute of Animal Pathology, University of Bern, Bern, Switzerland; 4 Center for Immunity and Inflammation, New Jersey Medical School, Newark, New Jersey, United States of America; 5 Department of Human Genetics, Leiden University Medical Center, Leiden, The Netherlands; NIAID/NIH, United States of America

## Abstract

Approximately one-third of the world's population suffers from chronic helminth infections with no effective vaccines currently available. Antibodies and alternatively activated macrophages (AAM) form crucial components of protective immunity against challenge infections with intestinal helminths. However, the mechanisms by which antibodies target these large multi-cellular parasites remain obscure. Alternative activation of macrophages during helminth infection has been linked to signaling through the IL-4 receptor alpha chain (IL-4Rα), but the potential effects of antibodies on macrophage differentiation have not been explored. We demonstrate that helminth-specific antibodies induce the rapid trapping of tissue migrating helminth larvae and prevent tissue necrosis following challenge infection with the natural murine parasite *Heligmosomoides polygyrus bakeri* (*Hp*). Mice lacking antibodies (J_H_^−/−^) or activating Fc receptors (FcRγ^−/−^) harbored highly motile larvae, developed extensive tissue damage and accumulated less Arginase-1 expressing macrophages around the larvae. Moreover, *Hp*-specific antibodies induced FcRγ- and complement-dependent adherence of macrophages to larvae *in vitro*, resulting in complete larval immobilization. Antibodies together with helminth larvae reprogrammed macrophages to express wound-healing associated genes, including Arginase-1, and the Arginase-1 product L-ornithine directly impaired larval motility. Antibody-induced expression of Arginase-1 *in vitro* and *in vivo* occurred independently of IL-4Rα signaling. In summary, we present a novel IL-4Rα-independent mechanism of alternative macrophage activation that is antibody-dependent and which both mediates anti-helminth immunity and prevents tissue disruption caused by migrating larvae.

## Author Summary

Intestinal helminths present a pressing problem in developing countries with approximately 2 billion people suffering from chronic infection. To date no successful vaccines are available and a detailed mechanistic understanding of anti-helminth immunity is urgently needed to improve strategies for prevention and therapy. Antibodies form a crucial component of protective immunity against challenge infections with intestinal helminths. However, the exact mechanisms by which antibodies target these large multi-cellular parasites have remained obscure. We now demonstrate that helminth-specific antibodies induce the rapid trapping of tissue migrating helminth larvae by activating phagocytes. In the absence of antibodies or their receptors, helminth-infected mice developed extensive tissue damage, revealing a novel role for antibodies in limiting parasite-caused tissue disruption. Furthermore, helminth-specific antibodies reprogrammed macrophages to express wound-healing factors such as the arginine-metabolizing enzyme Arginase-1. Interestingly, the Arginase-1 product L-ornithine directly impaired the motility of helminth larvae. In summary, our study provides detailed mechanistic insights into how antibodies can modulate phagocyte function to provide protection against a large multi-cellular parasite. Our findings suggest that novel anti-helminth vaccines should target the larval surface and activate wound-healing macrophages to provide rapid protection against tissue-disruptive larvae.

## Introduction

Intestinal helminths present a major global health burden, particularly in developing countries. Patients that are infected with nematodes such as *Ascaris lumbricoides*, *Trichuris trichuria* or *Necator americanus* often develop severe pathology and impaired responsiveness to vaccines [1] [2] [3] [4]. Approximately 2 billion people are infected with intestinal nematodes, with the most severe infections often found within school children [5] [6]. Although such infections can be treated by chemotherapy, worm burdens typically reach pretreatment levels within 6 months [7] [5]. Moreover, drug resistant helminths present a pressing problem for livestock [8], raising concerns about the long-term validity of chemotherapy amongst human populations [9] [10] [11] [6] [12] [13]. Unfortunately, no efficacious vaccines against intestinal nematodes are available to date, making an improved understanding of host immunity imperative.

Macrophages are highly plastic immune cells that can fulfill diverse tasks in immunity, metabolism and wound-healing depending on their tissue location and inflammatory context [14]. In the context of bacterial infection, the activation of classically activated macrophages by serum components such as antibodies and complement can enhance the phagocytosis and killing of bacterial or fungal pathogens [15]. By contrast, type 2 immune responses associated with helminth infection and allergies are characterized by a predominance of alternatively activated macrophages (AAM) that appear to play a role in anti-helminth immunity and wound repair through ill-defined mechanisms [16] [17].

Infection with the natural murine parasite *Heligmosomoides polygyrus bakeri* (*Hp*) is a common model used to study immunity against helminth infection [18]. Following primary (1°) infection with *Hp*, C57BL/6 mice develop a chronic infection [19]. In contrast, *Hp* fails to establish chronicity after challenge infection, largely due to the rapid development of a protective T_H_2 type granuloma around the tissue invasive larvae [20] [21] [22]. The highly concentrated accumulation of Arginase-1 (Arg1) expressing alternatively activated macrophages in inflammatory lesions is a hallmark of type 2 responses associated with allergy or helminth infection [23] [24] [25]. Recent reports have demonstrated an important role for type 2 cytokine driven alternative activation of macrophages in protective immunity against intestinal helminth infection [16] [26] [27]. Previous work [28] [29], including a study from our own group [30], additionally identified helminth-specific antibodies as essential components of immunity against *Hp*. Passive transfer of IgG or immune serum could also confer resistance to *Hp*, *Ascaris suum* and *Strongyloide*s *ratti* [31] [32] [33], and antibody production has been found to correlate with protection in human helminth infection [34] [35] [36]. Whilst antibodies contribute to protective immunity against *Hp* and other nematodes such as *Trichuris muris* or whipworms such as *Trichinella spiralis*, which cause chronic infections [37] [38], they are not required for rapid expulsion of the hookworm *Nippostrongylus braziliensis* [29]. However, the mechanism of antibody-mediated immunity against intestinal helminths has remained obscure.

In the current study we investigated the mechanisms of antibody-mediated immunity following challenge *Hp* infection and demonstrate that antibodies function to trap helminth larvae and to prevent parasite-induced tissue damage. Using newly developed tools for image analysis, we show that antibody-activated macrophages upregulate Arg1 and immobilize infective and tissue dwelling larvae. Intriguingly, the reprogramming of macrophages by helminth-specific antibodies did not require IL-4Rα signaling, indicating that antibody activation of macrophages during helminth infection represents a novel pathway of alternative macrophage differentiation. In summary we have shown that antibodies in the presence of helminth antigens can elicit a novel subtype of IL-4Rα independent alternatively activated macrophages, which we refer to as helminth-antibody activated macrophages (HAAM).

## Results

### Mice deficient in antibodies or activating antibody receptors exhibit impaired early immunity to *H. polygyrus bakeri* challenge infections

**Figure 1:**
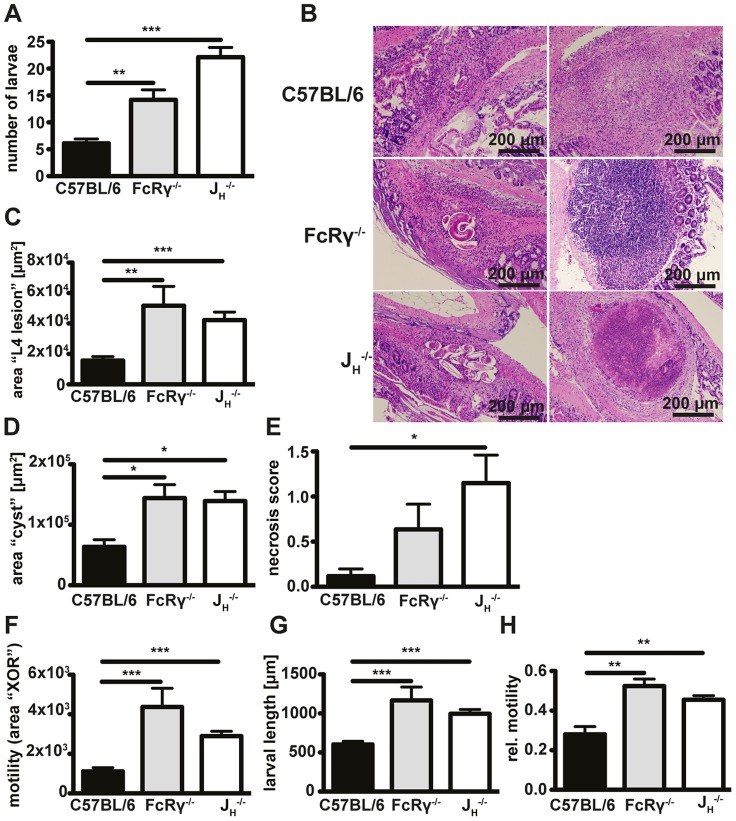
Antibody and FcRgamma-chain deficient mice show increased larval burdens, larger granulomas, a stronger tendency to develop necrosis and higher in-tissue motility as compared to wildtype mice. Mice were challenge infected with 200 infective L3 larvae after drug cured 1° infection and sacrificed on day 4 p.i.; (A) Numbers of tissue invasive larvae were analysed with a modified Baermann apparatus. (B) Paraffin sections of the duodenum of infected mice were hematoxylin and eosin (H&E) stained, (C/D) granuloma areas were quantified in light microscopy images of the largest cross-section of serial sections with larvae (C) or without larvae (D). (E) Necrosis in H&E-stained sections was scored by a blinded pathologist. (F–H) Absolute (F) and relative (H) in-tissue motility or larval length (G) was assessed by light microscopy combined with our Fiji macro. Pooled data of two independent experiments with 5 mice per group are shown as mean + SEM (*p<0.05, **p<0.01, ***p<0.001, Mann-Whitney test).

Our previous work on antibody mediated protective immunity against *Hp* has largely been focused on late time points (day 14–20) after challenge infection, when adult worms can be found in the lumen of the small intestine [30]. However, protective immunity is initiated as early as day four of challenge *Hp* infection [16] [29], when larvae have first invaded the intestinal mucosa. Thus, in order to investigate a potential early effect of antibodies in the memory response to *Hp*, we analysed the numbers of larvae that had invaded the small intestine of infected antibody-deficient J_H_^−/−^ mice (for accession numbers of gene name abbreviations used in this manuscript see Table 1). J_H_^−/−^ mice carry a deletion of the joining fragment of the immunoglobulin heavy chain locus and are devoid of mature B-cells and antibodies [39]. As shown in Fig. 1A, and in line with previous work [29], J_H_^−/−^ harbored significantly higher numbers of L4 on day 4 after challenge infection. As previous work indicated a small but significant contribution of cellular activation via activating Fc receptors on the numbers of adult worms [30], we additionally assessed numbers of larvae in challenge infected mice lacking activating Fc receptors (FcRγ^−/−^). Interestingly, these mice also exhibited clearly increased larval burdens (Fig. 1A).

**Table 1:** Accession numbers for abbreviations of gene name used in the text.

Gene name	Accession number (UniProt)
Arg1	Q61176
C3	P01027
CD11b	A1E2I0
CD16	P08508
CD64	P26151
CD32	P08101
CXCL2	Q6PUJ1
CXCL3	Q6W5C0
FcRγ	Q8AY96
IL4	P07750
IL4Rá	P16382
IL13	20109
IL33	Q8BVZ5
Jag1	Q9QXX0
J_H_	Q9QXF0

### Antibody and FcRgamma-deficient mice exhibit larger granulomas, a tendency to develop necrosis and greater larval motility

In addition to the increased numbers of larvae, we observed that J_H_^−/−^ and FcRγ^−/−^ mice exhibited larger granulomas at day 4 post challenge *Hp* infection. Assessment of hematoxylin and eosin (H&E) stained tissue sections revealed that granulomas in all sets of mice were characterized by epitheloid macrophages and eosinophils and often contained cuticle remains or intact larvae (Fig. 1B). Quantification of the inflamed area for lesions containing intact larvae (Fig. 1B, left column), or for lesions without intact larvae (Fig. 1B, right column), showed that J_H_^−/−^ and FcRγ^−/−^ mice had indeed developed more extensive granulomas around intact larvae (Fig. 1C) and larger “cysts” without visible larvae (Fig. 1D). Finally, pathological scoring of H&E stained granuloma sections showed an increased tendency to develop necrosis in both J_H_^−/−^ and FcRγ^−/−^ mice (Fig. 1E).

We rationalized that a failure to efficiently trap larvae in the granuloma might contribute to the observed increase in tissue necrosis. As the assessment of larval numbers by a modified Baermann apparatus only gives an indirect measure of larval motility [16], we quantified in-tissue motility directly by microscopy. Our analysis of time-lapse movies (Text S1, Fig. S1) showed that in-tissue motility was increased in the absence of antibodies or activating antibody receptors (Movie S1, Fig. 1F). During our analysis, we also noted pronounced differences in the size of larvae between wildtype, FcRγ^−/−^ and J_H_^−/−^ mice (Fig. 1G). We therefore calculated the difference in the larval position after 60s relative to the total larval area. In FcRγ^−/−^ and J_H_^−/−^ mice, the movement of the larvae was still observed to be greater than that seen for wildtype mice even when the movement was normalized to larval size (Fig. 1H).

### Cellular compositions of granulomas are similar in wildtype and antibody-deficient mice

Due to the apparent involvement of cellular activation via Fc receptors in antibody-mediated protection against *Hp* (Fig. 1) we performed a flow cytometric analysis of granuloma cell populations, with a focus on macrophages and granulocytes as major FcR expressing cells. As the absence of antibodies resulted in a defective immune response already at day 4 post challenge infection, we compared granuloma cell populations from challenge infected J_H_^−/−^ and wildtype mice by flow cytometry using the gating strategy described in Fig. S2A. No significant differences in the cellular infiltrate were noted between the two strains, with the exception of basophils that were absent from the granuloma of antibody-deficient mice (Fig. S2B). The absence of basophils observed in J_H_^−/−^ mice was in keeping with our previous work, showing an important role for antibodies in basophil expansion during *Hp* infection [40]. However, basophils represent a minor cell population in the granuloma and basophil depletion during challenge infection only had a minor impact on protective immunity [40]. Thus, we concluded that antibody-FcRγ-chain-mediated protection is likely to involve other cell types.

### Granuloma macrophages express high levels of antibody receptors and efficiently bind IgG1 and IgG3

**Figure 2:**
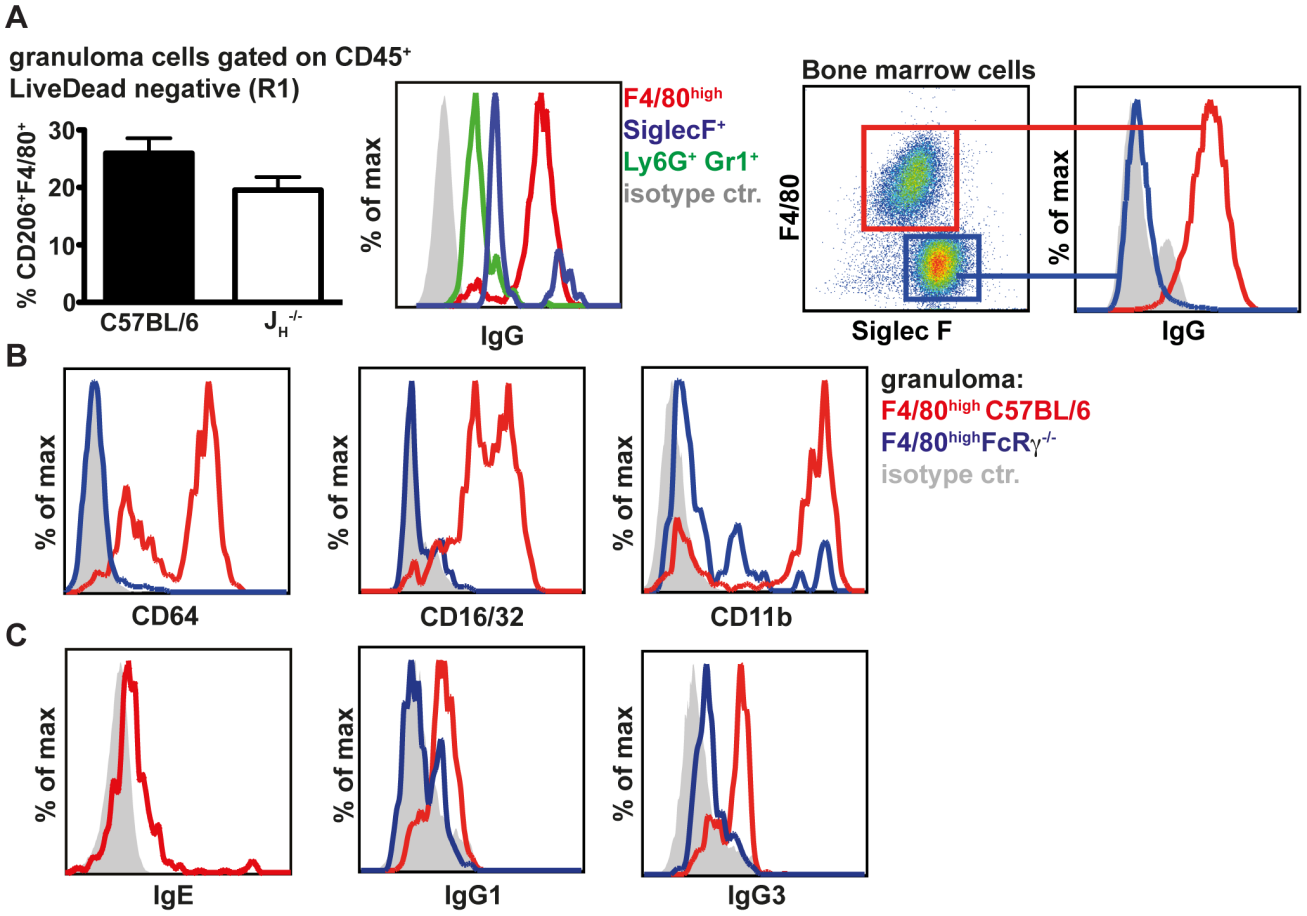
Macrophages are the major IgG^high^ population in the granuloma following challenge *Hp* infection and express high levels of antibody and complement receptors. (A) 25–30 granuloma were excised from the small intestine of challenge *Hp* infected mice on day 4 p.i. and isolated granuloma cells (left panels) or immune serum activated bone marrow derived cells (right panels) were stained for cell surface markers and IgG. (B) Expression of CD64, CD16/32 and CD11b on granuloma macrophages from C57BL/6 or FcRγ^−/−^ mice; (C) Levels of IgE, IgG1 and IgG3 on granuloma macrophages from C57BL/6 or FcRγ^−/−^ mice; Representative FACS plots from two independent experiments with 4–5 mice per group are shown.

The similarities in the cell recruitment between wildtype and antibody deficient mice suggested that antibodies might differentially activate resident or recruited cells at the site of infection. As our previous data indicated a predominant role of IgG [30] we analysed surface IgG on macrophages (F4/80^high^, Ly6G^−^), eosinophils (SSC^high^, SiglecF^+^) and neutrophils (Ly6G^+^ Gr1^+^) by flow cytometry. Our analysis showed that macrophages, which were the most abundant cell type in day 4 granulomas, displayed the highest surface levels of IgG, with eosinophils exhibiting moderate binding and neutrophils very little binding (Fig. 2A, left panel). We then confirmed the different IgG binding capacities of macrophages and eosinophils *in vitro* with bone marrow derived cells incubated with *Hp* immune serum (Fig. 2A, right panel). Further characterization of granuloma macrophages by flow cytometry showed high surface expression of activating FcγRs (CD64 and CD16) as well as CD11b, which is involved in complement-mediated immune complex binding [41] (Figs. 2B, S2C). In addition, macrophages displayed low levels of IgE but considerable levels of IgG1 and IgG3 on their surface (Fig. 2C).

When comparing granuloma macrophages in challenge *Hp* infected wildtype and FcRγ^−/−^ mice, we observed that FcRγ^−/−^ mice displayed reduced levels of surface IgG1 and IgG3 (Figs. 2C, S2C). Interestingly CD11b levels were also reduced on granuloma macrophages of FcRγ^−/−^ mice (Fig. S2C). Thus, in addition to the engagement of activating Fc receptors, helminth-antigen-antibody immune complexes might bind to CD11b in a complement dependent manner [42] [43] [44].

### Antibodies act to upregulate Arginase-1 expression by macrophages *in vitro* and *in vivo*

We next investigated potential differences in the activation of macrophages in the presence or absence of specific antibodies following challenge infection with *Hp*. As RNA samples from granulomas were not of sufficient quality for microarray analysis, we performed *in vitro* experiments with bone marrow-derived macrophages (BMMac) that we incubated with *Hp* immune serum (1∶50, v∶v) and infective *Hp* L3 larvae (500 larvae/10^6^ cells). Gene expression was compared using microarray analysis of macrophages incubated with larvae alone or with larvae in combination with immune serum. The microarray analysis identified a total of 216 genes that were differentially expressed (up- or down- regulated more than 1.5 fold). Among the ten most downregulated genes, we identified several T_H_1 associated genes such as interleukin 12b (*IL12b*), interferon regulatory factor 1 (*Irf1*) and interferon inducible GTPase 1 (*Iigp1*). However for our further experiments, we focused on the ten genes that were found to be upregulated more than two-fold by the combination of immune serum and larvae. Of note, these included several factors involved in tissue repair processes such as angiogenesis, cell proliferation, and remodeling as well as granulocyte recruitment and activation, or type 2 immunity (Table 2). Subsequent qPCR analysis confirmed the significant up-regulation of *CXCL3*, *CXCL2*, *IL-33*, *Jag1* and *Arg1* (Figs. S3, 3A). However, *Arg1* was the only gene that was upregulated to a lesser extent in macrophages from FcRγ^−/−^ mice. Moreover, *Arg1* was moderately upregulated when BMMac were stimulated with larvae and purified 2° IgG (100 µg/ml), which could be greatly enhanced, when naïve serum was added together with 2° IgG and larvae (Fig. S3J). Hence, helminth-specific IgG can reprogram macrophages and this effect is amplified by the presence of complement components.

**Figure 3:**
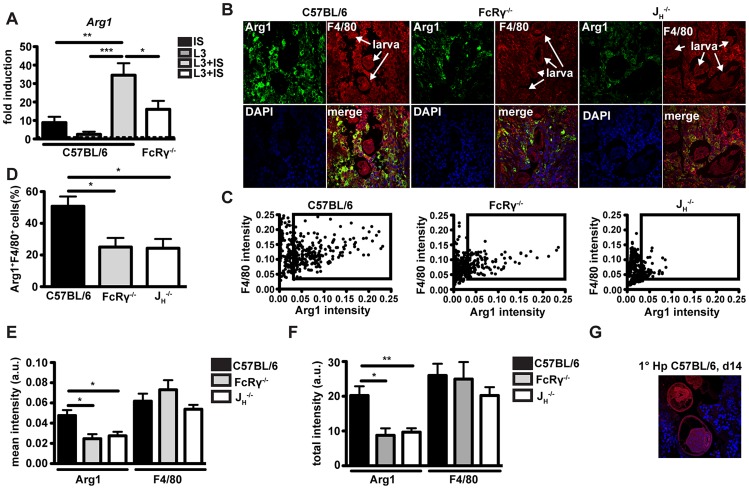
*Arg1* is induced by immune serum and *Hp* larvae *in vitro* and Arg1 expressing macrophages are less abundant in the granulomas from antibody and FcRgamma-chain deficient mice. (A) *Arg1* expression in BMMac from C57BL/6 or FcRγ^−/−^ mice cultured in the presence or absence of larvae and/or immune serum was quantified by qPCR. (B) Paraffin sections of the upper duodenum of challenge *Hp* (day 4 p.i.) infected C57BL/6, FcRγ^−/−^ or J_H_^−/−^ mice were immune-fluorescently stained for Arg1 (green), F4/80 (red) and nuclei (blue), representative pictures are shown. (C–F) Intensities of Arg1 and F4/80 were quantified using Fiji and Cell Profiler software. (C) Scatter plots of intensities for F4/80 and Arg1 for all detected cells in the region of interest (ROI) (D) Frequency of Arg1^+^ F4/80^+^ (intensity >0.035) cells in the ROI, (E) Mean intensity of Arg1 and F4/80 staining in all detected cells in the ROI (F) Total intensity of Arg1 and F4/80 staining per ROI; Pooled data from two independent experiments with 3–6 mice per group are shown as mean + SEM (*p<0.05, **p<0.01); (G) Arg1^+^ macrophages are absent from the granuloma of 1° *Hp* infected mice (day 14 p.i.), representative picture.

**Table 2:** Fold induction and function of the 10 most upregulated genes in macrophages co-cultured with immune serum in combination with larvae as compared to larvae alone.

Gene name	Fold change	Function (with relation to macrophages)
***CXCL3***	**8.7**	Granulocyte recruitment & activation [74], angiogenesis [75]
***Jag1***	**2.6**	Angiogenesis [76], T_H_ cell differentiation [77]
*Emp2*	2.6	Cell adhesion [78], angiogenesis [79]
*Trem1*	2.5	Induction of immune responses [80],[81], inflammatory response to S*chistosoma* [82]
*Tpbpa*	2.4	Placental function & maternal vasculature remodeling [83]
***CXCL2***	**2.4**	Granulocyte recruitment & activation [74]
*S100A8*	2.3	Stress response [84], immune regulation & wound healing [85]
***Arg1***	**2.3**	Wound healing, immune regulation [46], immunity to helminths [16]
***Il33***	**2.2**	Induction of T_H_2 response [86] (activation of basophils, eosinophils, T_H_2 cells)
*Inhba*	2.1	Cell growth [87], T_H_2 cytokine & inducer of alternative activation [88]

Bone marrow derived macrophages (10^6^/ml) were cultured with larvae (500/ml) in the presence or absence of immune serum. Cells were detached from plates or larvae and passed through 40 µm filters before collection for RNA extraction. Changes in gene expression were identified by whole mouse genome microarray (Affymetrix) and confirmed by qPCR (for genes in bold). For a description of the microarray analysis, see Text S1.

We next investigated the impact of antibodies on Arg1 protein expression by macrophages *in vivo*. Arg1 expression in granuloma sections from wildtype, J_H_^−/−^ or FcRγ^−/−^ mice was analysed by immunofluorescence and confocal microscopy (Fig. 3B). In keeping with our gene expression data, granulomas of antibody-deficient mice harbored significantly lower numbers of Arg1^high^ macrophages (Fig. 3B). Quantification of the intensities of the Arg1 and F4/80 staining for each cell resulted in scatter plots, which clearly showed that most F4/80^+^ cells expressed Arg1 in the granuloma of C57BL/6 but not of FcRγ^−/−^ or J_H_^−/−^ mice (Fig. 3C). Furthermore, significant differences were noted not only for the frequency of Arg1^high^ macrophages (Fig. 3D) but also for the mean Arg1 intensity in all detected cells (Fig. 3E) and the total Arg1 intensities in the region of interest (Fig. 3F). Of note, we could not detect any Arg1 expressing cells in the granuloma of 1° *Hp* infected C57BL/6 mice (Fig. 3G), indicating that effector mechanisms that arise only following challenge infection are required for Arg1 upregulation.

### Immune serum from challenge-infected mice induces the adherence of macrophages to *Hp* larvae *in vitro*

**Figure 4:**
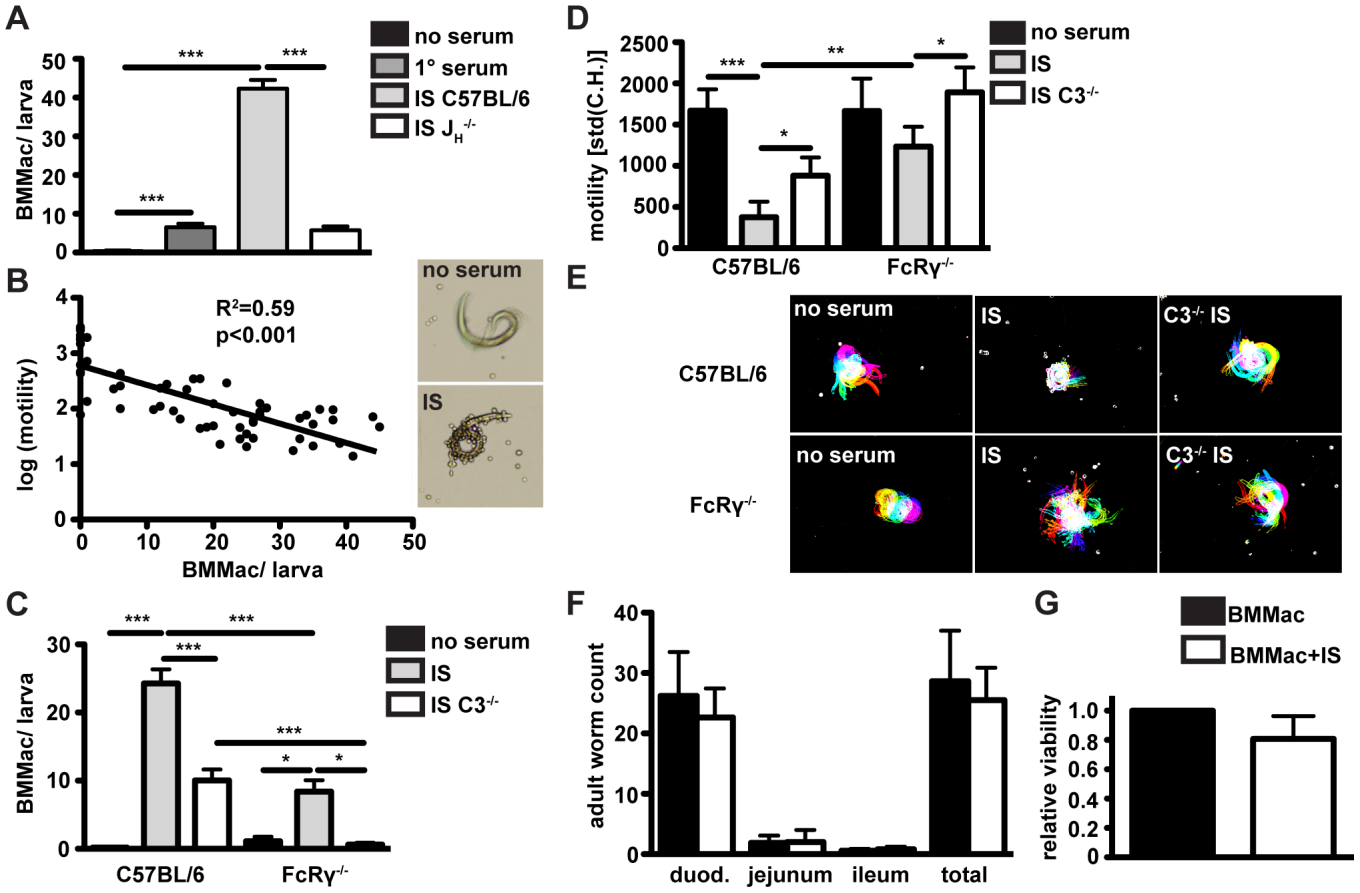
Antibodies from challenge *Hp* infected wildtype mice induce adherence of bone marrow derived macrophages to *Hp* larvae *in vitro*, causing larval immobilization dependent on FcRgamma-chain and complement component 3; Larval infectivity and viability is not affected by adhering macrophages. (A) BMMac from C57BL/6 mice were co-cultured with larvae in the presence or absence of serum from 1° or challenge *Hp* infected C57BL/6 or J_H_^−/−^ mice, adherent macrophages per larva were counted in light microscopy images. (B) Correlation of the number of adhering BMMac and larval motility, (C/D) C57BL/6 or FcRγ^−/−^ BMMac were cultured with larvae in the presence or absence of immune serum from C57BL/6 or FcRγ*C3^−/−^ mice. Adherent macrophages per larva were counted in light microscopy images (C) and larval motility was quantified by Fiji (D). (E) Representative time lapse pictures, (F) Larval infectivity was analysed by infecting C57BL/6 wildtype mice with 100 larvae recovered from BMMac co-cultures in the presence or absence of immune serum. Adult worms in the three parts of the small intestine were counted on day 10 p.i.. (G) The larval viability relative to control (cultured without BMMac) after co-culture with or without immune serum was quantified by ATP-assay. Pooled data from three independent experiments with bone marrow from 2–3 mice or with 5 mice per group are shown as mean + SEM (*p<0.05, **p<0.01, ***p<0.001, Mann-Whitney test).

In our *in vitro* co-cultures of bone marrow derived macrophages (BMMac) and larvae, we observed that immune serum from challenge *Hp* infected C57BL/6 mice (collected at day 4 p.i.) induced the adherence of macrophages to larvae (Fig. 4A). By contrast, serum from 1° *Hp* infected C57BL/6 or challenge *Hp* infected J_H_^−/−^ mice showed an attenuated capacity to induce macrophage adherence (Fig. 4A). Immune serum-induced adherence could be confirmed using macrophages from the peritoneal cavity of naïve mice (Fig. S4A), or using BMMac of a different genetic background (Balbc) (Fig. S4B). As IgG^+^ eosinophils were also present in the granuloma, we additionally tested the ability of these cells to adhere to larvae. Bone marrow-derived eosinophils activated with immune serum hardly adhered to larvae, and addition of these cells to macrophage cultures decreased rather than increased macrophage adherence (Fig. S4B). These data indicate that *Hp*-specific antibodies predominantly act on macrophages to promote cellular adherence to larvae.

To rule out a possible contribution of IL-4 from immune serum we determined the role of this cytokine in macrophage adherence. Addition of IL-4 to macrophage larvae co-cultures did not alter immune serum-induced macrophage adherence and larval immobilization (Fig. S5A) or larval fitness (Fig. S5B), and levels of IL-4 or IL-13 present in the immune serum or macrophage-larvae co-culture supernatants were all below the ELISA detection limit (Figs. S5C, D).

### Macrophages immobilize *Hp* larvae in an antibody-, FcγR- and complement-dependent manner

We also noted that larvae covered in helminth-antibody activated macrophages exhibited a striking reduction of larval motility. To analyze this in a quantitative manner we developed a method to analyze larval motility in time-lapse movies. This analysis showed a clear correlation between the number of adherent macrophages per larvae and the reduction in larval motility, measured as the difference in the larval shape between consecutive frames of time-lapse movies (Fig. 4B) (detailed description Fig. S1B–D). We next explored the role of activating FcRs and complement in immune serum-induced macrophage adherence to *Hp* larvae. When co-cultured with larvae in the presence of immune serum, FcRγ^−/−^ macrophages adhered less efficiently to larvae as compared to wildtype macrophages (Fig. 4C). Using BMMac from mice lacking all IgG receptors (FcγRI/II/III/IV^−/−^), we confirmed the important role of IgG for efficient trapping of larvae (Fig. S4E). As expected, FcγRI/II/III/IV^−/−^ displayed no detectable surface IgG, CD16/32 (FcγRII/III) or CD64 (FcγRI) and both FcγRI/II/III/IV^−/−^ and C57BL/6 BMMac did not show IgM binding (Fig. S4F). To rule out a possible role of IgE for larval trapping, we studied macrophage adherence and larval immobilization, using immune serum from challenge infected IgE^−/−^ mice. IgE-deficient immune serum potently induced BMMac adherence as well as larval immobilization (Figs. S4G, H).

As for FcRγ^−/−^ mice, our previous work had shown a small but significant contribution of complement to reducing adult worm numbers following challenge *Hp* infection [30]. Thus to determine a possible contribution of complement in larval immobilization we used complement component 3 (C3)-deficient immune serum in our macrophage assays. C3-deficient immune serum had a clearly reduced ability to promote macrophage adherence to wildtype macrophages and adherence of macrophages from FcRγ^−/−^ mice was completely abolished (Fig. 4C). Moreover, FcRγ^−/−^ macrophages had a lower capacity to trap larvae in the presence of wildtype immune serum, and failed completely to immobilize larvae when incubated with C3-deficient immune serum (Figs. 4D/E) (Movie S2). These data indicate that immune serum acts to promote macrophage adherence and larval immobilization in a manner involving the contribution of activating FcRs and complement.

Of note, immune serum also triggered adherence of BMMac to tissue dwelling L4 stage larvae, which we recovered from the small intestine of infected mice. Although L4 larvae were less motile than infective L3 larvae, immune-serum induced macrophage adherence also had a negative effect on their motility *in vitro* (Movie S3).

In order to study a potential effect of immune serum-induced macrophage adherence on larval fitness, we next infected mice with larvae recovered from immune serum-supplemented macrophage co-cultures. Adult worm counts on day 10 p.i. indicated that larvae cultured with BMMac and immune serum for 24h were still as infective as larvae cultured with macrophages alone (Fig. 4F). As another measure of larval fitness, we quantified ATP levels in larval homogenates following their recovery from macrophage co-cultures. Again we could not detect any significant difference in the viability after culture with BMMac alone, or with a combination of immune serum and BMMac (Fig. 4G). Taken together these data suggest that helminth-antibody-dependent macrophage activation promotes their adherence to result in an impact on larval motility but not on larval viability.

### Absence of Arginase-1 reduces immune serum-induced larval trapping

**Figure 5:**
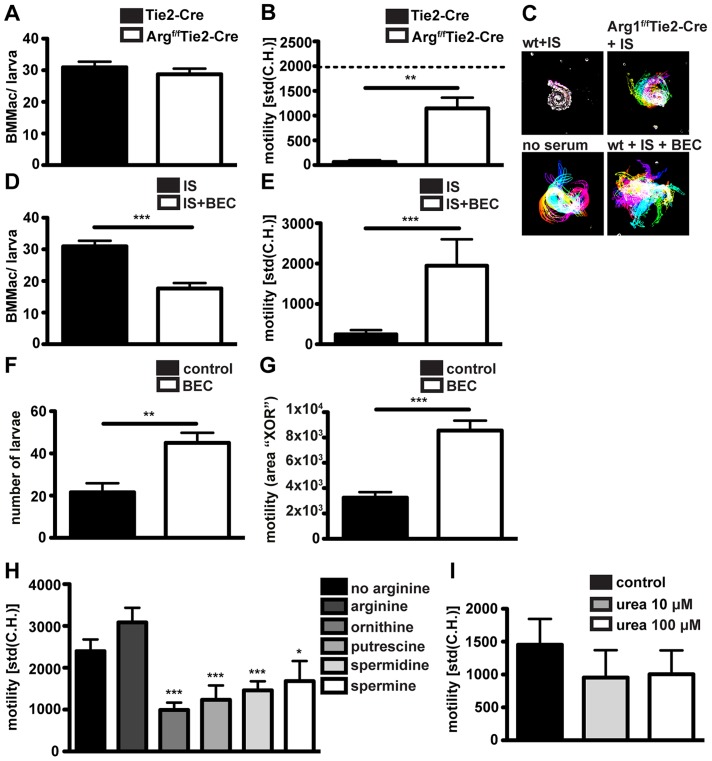
Arg1 is needed for to efficient larval trapping *in vitro* and *in vivo* and L-ornithine or polyamines but not urea can reduce larval motility *in vitro*. (A/B) BMMac from Tie2-Cre or Arg^f/f^Tie2-Cre mice were co-cultured with larvae in the presence or absence of immune serum from C57BL/6 mice and adherence (A) and motility (B) were assessed by light microscopy. Dashed line in panel B depicts motility of larvae in the presence of BMMac without addition of immune serum. (C) Representative time lapse pictures; (D/E) Adherence and motility of C57BL/6 BMMac in the presence of the Arg1 inhibitor BEC (1 µM); (F/G) C57BL/6 mice were treated with BEC (0.2%) during challenge infection and larval motility (F) and numbers (G) were analysed; (H/I) Larvae were cultured in L-arginine free medium with or without supplementation with L-arginine (570 µM) +/− L-ornithine, putrescine, spermidine or spermine (each 100 µM) (H) or urea (I) and motility was quantified. Pooled data from two independent experiments (3–6 mice per group) are shown as mean + SEM (*p<0.05, **p<0.01, ***p<0.001, Mann-Whitney test).

Our previous data demonstrated that antibody-FcγR interactions upregulated *Arg1* gene expression in macrophages co-cultured with larvae *in vitro*, and were necessary for Arg1 protein expression by granuloma macrophages *in vivo*. Moreover Arg1 has previously been reported to contribute to protective immunity against *Hp* [16]. We therefore investigated a possible contribution of this enzyme to macrophage adherence and larval immobilization. We performed *in vitro* co-cultures of bone marrow-derived macrophages from Arg1^f/f^Tie2-Cre [45] [46] or Tie2-Cre [47] mice with *Hp* larvae in the presence of immune serum. The Tie2-Cre deleter was originally reported to result in Cre recombinase mediated gene ablation in endothelial cells [47], but was later discovered to show robust (80–90%) recombination also in hematopoietic cells [48]. We confirmed Arg1^f/f^ and Tie2-Cre transgene expression and partial deletion of Arg1 in Arg1^f/f^Tie2-Cre mice (Figs. S5H–J). We could not observe any difference in the capacity of Arg1^f/f^Tie2-Cre and wildtype Arg1Tie2-Cre macrophages to adhere to larvae (Fig. 5A). However, Arg1^f/f^Tie2-Cre macrophages had a reduced ability to immobilize larvae after incubation with immune serum (Figs. 5B, C) (Movie S4). To further confirm the role of Arg1 in antibody- mediated larval trapping, we added the Arg1 inhibitor S-(2-boronoethyl)-L-cysteine (BEC) to co-cultures of BMMac and larvae in the presence of immune serum. Addition of BEC *in vitro* resulted in reduced macrophage adherence and clearly impaired larval immobilization (Figs. 5, E).

Finally, we tested the relevance of Arg1 in larval trapping *in vivo*, by treating challenge-infected C57BL/6 mice with BEC (0.2%, oral gavage, once daily). In keeping with previous findings [16], BEC treatment resulted in higher larval burdens (Fig. 5F). Moreover, larvae in BEC treated mice showed higher in-tissue motility (Fig. 5G, Movie S5).

Arg1 catalyzes the breakdown of L-Arginine into urea and L-ornithine, and BMMac produced considerable amounts of urea when activated with immune serum and *Hp* larvae (Fig. S5F). We first investigated a potential effect of L-arginine depletion by culturing larvae in L-arginine-free medium. However, we did not observe significant changes in the larval motility in the absence of L-arginine (Fig. 5H). As larvae are surrounded by large numbers of Arg1 expressing macrophages in challenge *Hp* infected mice, trapped larvae are likely to be exposed to high levels of Arg1 products. To replicate this process *in vitro* we incubated larvae with L-ornithine or polyamines (putrescine, spermidine and spermine) or urea. While urea did not significantly affect larval motility (even at 100 µM) (Fig. 5I), L-ornithine and polyamines clearly impaired larval movement (Fig. 5H, Movie S6). These data implicate L-arginine metabolism to polyamines as a possible mechanism by which Arg1 contributes to protective immunity against *Hp*.

### Antibodies can induce Arg1 independently of IL-4Rα signaling

**Figure 6:**
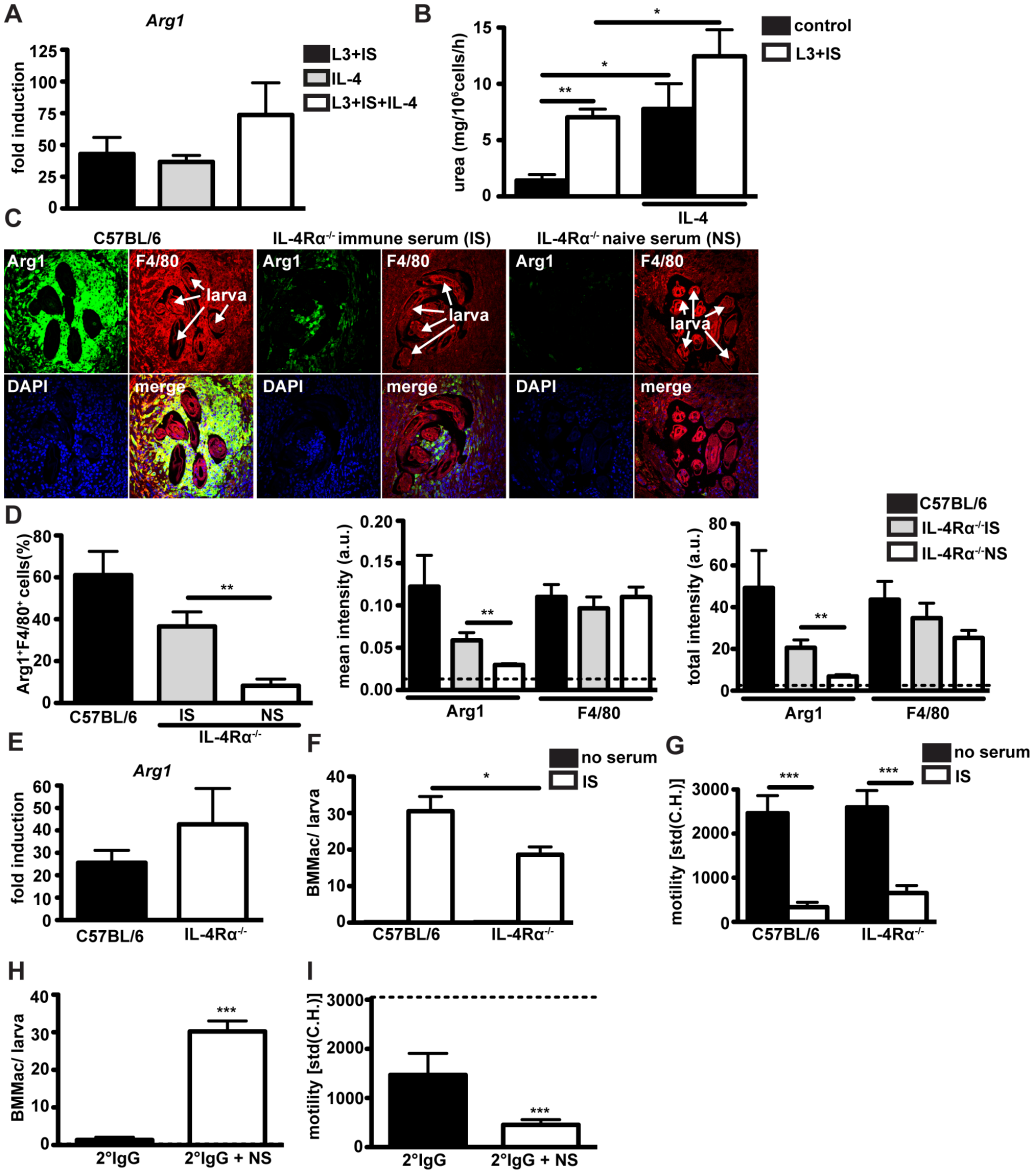
Antibodies can induce Arg1 and larval trapping by macrophages independently of IL-4Ralpha signaling. (A) *Arg1* expression in BMMac treated with larvae and immune serum and/or IL-4 or was quantified by qPCR and calculated relative to untreated control (B) Urea production in macrophages (treated as in (A)) was quantified after performing an Arg1 activity assay. (C) Sections of the small intestine from challenge-infected C57BL/6 or IL-4Ralpha^−/−^ mice, treated with immune or naïve serum were stained for Arg1 (green) and F4/80 (red) and counterstained with DAPI (blue). Representative pictures from two independent experiments with 3–4 mice per group are shown. (D) Frequencies of Arg1^+^F4/80^+^ cells, mean intensities or total intensities of Arg1 and F4/80 were quantified using Fiji and Cell Profiler software. Dashed line depicts background fluorescence (E). *Arg1* expression in wildtype or IL-4Ralpha^−/−^ BMMac treated with larvae and immune serum was quantified by qPCR. (F/G) BMMac adherence (F) and larval motility (G) were analysed after co-culture with larvae in the presence or absence of immune serum. (H/I) Adherence (H) and motility (I) after treatment with larvae and purified 2° IgG −/+ naïve serum (NS). Dashed line depicts motility of larvae with BMMac alone. Pooled data from two independent experiments with 3–4 mice per group are shown as mean + SEM (*p<0.05, **p<0.01, ***p<0.001, Mann-Whitney test).

IL-4 has long been recognized as a major factor responsible for the upregulation of Arg1 [49]. Thus, we analysed *Arg1* expression in BMMac after culture with larvae and immune serum in the presence of IL-4. IL-4 alone clearly induced *Arg1* to similar levels as observed for immune serum (Fig. 6A), and the combination of IL-4 plus immune serum resulted in a slightly stronger Arg1 induction, both on the mRNA and enzymatic activity level (Figs. 6A, B).

Arg1 expression during helminth infection is normally associated with an AAM phenotype dependent upon IL-4Rα signaling [49] [16] [50]. Hence, we further investigated the role of IL-4Rα signaling on antibody-induced upregulation of Arg1 following challenge *Hp* infection. As IL-4Rα^−/−^ mice exhibit a strongly impaired antibody response during primary and challenge infections with *Hp* [40], we treated challenge-infected mice with immune serum (collected at day 4 p.i.) from immune C57BL/6 mice. As shown in Fig. 6C, IL-4Rα^−/−^ mice showed reduced numbers of F4/80^+^ macrophages surrounding tissue dwelling larvae. However, treatment with immune serum did result in an increase in Arg1^+^ macrophages within the granuloma of IL-4Rα^−/−^ mice, indicating that *Hp*-specific antibodies can induce Arg1 expression *in vivo* independently of IL-4Rα signaling (Figs. 6C, D). To confirm that the ability of antibodies to trigger *Arg1* expression in macrophages occurs independently of IL-4Rα signaling, we performed co-cultures of IL-4Rα^−/−^ BMMac with *Hp* larvae. As shown in Fig. 6E, *Arg1* was potently upregulated in IL-4Rα^−/−^ BMMac in response to immune serum and larvae. Taken together these data indicate that IL-4 and antibodies can upregulate expression of Arg1 independently, but when present together will act in an additive manner.

Lastly, we investigated a potential role of IL-4Rα and other effector mechanisms that may contribute to immune serum-triggered larval trapping by macrophages. We observed that IL-4Rα^−/−^ BMMac displayed a significantly reduced capacity to adhere (Fig. 6F), but were still able to immobilize larvae (Fig. 6G). Furthermore, IgG antibodies purified from challenge immune serum promoted weak adherence and moderate larval immobilization, which could be greatly enhanced by naïve serum (Figs. 6H, I). These data suggest that helminth specific antibodies together with complement components can trigger larval trapping even in the absence of other effector molecules that may be present in challenge immune serum.

## Discussion

Numerous studies have documented a role for antibodies in providing protective immunity against helminths [34] [35] [36] [51] [37] [28] [30] [29], yet the mechanisms by which antibodies act against these large multicellular parasites have remained elusive. Our current work demonstrates a novel effector function of antibodies in activating macrophages to modulate the expression of genes normally associated with the alternatively activated phenotype. Antibody-mediated macrophage activation also triggered macrophage adherence to helminth larvae and resulted in a potent suppression of larval motility.

Interestingly, activating Fc receptors and complement C3 component acted together to activate macrophages and to limit larval motility *in vitro*. Crosstalk between the complement cascade and activating Fc receptors is well known to occur in autoimmunity [52]. We have previously shown that genetic ablation of either FcRγ-chain or the complement component C3 led to a significant but minor defect in the emergence of adult worms following challenge *Hp* infection between days 14–28. Our current data suggest that these two pathways act in a redundant and additive manner to promote antibody-induced larval trapping and to provide immunity against challenge *Hp* infections. The rapid activation of cells via Fc receptors is likely to be of particular importance during the early response (day 4) against the tissue dwelling larvae, as mice genetically deficient for FcRγ or B cells exhibited similar increases in larval motility and tissue necrosis within intestinal granulomas. The tissue dwelling stage of helminth larvae has been suggested to be a primary target of antibody-mediated protective immunity, which may be advantageous due to the immune-suppressive capacities of adult worms [53]. Our data further suggest that the antibody-mediated activation of granuloma macrophages mainly involves IgG1 and/or IgG3 isotypes, which may form immune complexes with helminth antigens or directly bind to the larval surface. As essential components of the larval cuticle are shared between different helminths, future work should delineate the specificities of protective antibody isotypes in order to identify potential vaccine targets.

Interestingly, although immune serum efficiently activated macrophages to immobilize larvae *in vitro*, it did not impact on larval viability. Given that the destruction of a large extracellular parasite such as *Hp* is likely to result in severe inflammation, the trapping and starvation of invasive larvae might be more beneficial for the host. In line with this view we observed that type 2-associated antibody production functioned to limit tissue necrosis and to induce tissue repair genes. Thus, type 2 immunity may act to limit larval migration through tissues, leading to a halt in the parasitic lifecycle and additionally serving to prevent excessive tissue damage. However we cannot rule out the possibility that other immune-mediated mechanisms also contribute to the killing of helminth larvae *in vivo*.

Of particular interest, arginase was required for the ability of helminth-antibody activated macrophages to inhibit larval motility as well as for efficient larval trapping *in vivo*. Arg1 expressing AAM are strongly associated with helminth infection [16] [54] [46] [55], and are typically regarded as controlling inflammation and tissue repair [46] [55] rather than as protective immune cells [56]. However, in keeping with our data Anthony et al. [16], previously reported a protective role for Arg1 expressing AAM in *Hp* infection. Our work expands on these findings to indicate that antibodies are essential for the robust Arg1 expression by macrophages following *Hp* infection. We also show that the Arg1 product L-ornithine and the polyamine metabolites putrescine, spermidine and spermine have a direct negative effect on larval motility, suggesting that excessive polyamines can impact on larval metabolism in a manner that interferes with motility. The finding that L-ornithine immobilizes larvae at concentrations that are below usual serum levels of this metabolite (around 50 µM) is surprising especially as granuloma may be expected to be a “leaky” environment. However, under homeostatic conditions, cell-free L-ornithine levels in the intestine might be relatively low due to the co-expression of Arginase 2 and L-ornithine metabolizing enzymes in enterocytes [57]. Thus, Arg1 activity might serve not only to control aberrant inflammation and fibrosis during parasite infection [46], but to also create a metabolically unfavorable environment for parasites. Future studies will be required to delineate the exact mechanism(s) by which polyamines affect larval motility.

Of note, IL-4Rα^−/−^ mice failed to accumulate the large numbers of macrophages that were observed in the granuloma of wildtype mice, indicating that IL-4Rα signaling is necessary for the recruitment and/or expansion of macrophages at the site of infection [16] [58]. Thus, even if macrophages could immobilize larvae in the absence of IL-4Rα signaling *in vitro*, IL-4Rα signaling might be essential for the accumulation of macrophages *in vivo*, which can then be activated by antibodies to immobilize tissue invasive larvae. This suggests that *in vivo*, antibodies and IL-4 may work together to elicit a potent activation of Arg1 expressing macrophages, which mediate protection. This might be due to the convergence of IL-4Rα and FcRγ-chain signaling at the level of downstream signaling events such as Spleen tyrosine (Syk) or PI3 kinases [59] [60] [61]. In a lung model of *Schistosom*a infection, Arg1 expression in the granuloma was found to be tightly concentrated around the egg despite abundant AAM and type 2 cytokines (IL-4 and IL-13) in the overall granuloma environment [25]. Thus, it is tempting to speculate that antibodies directed against helminth eggs or larvae can promote local Arg1 expression in areas of high antigen availability.

Previous publications have indicated that macrophages activated by type 2 cytokines express high levels of Arg1 [16] [49] [27], whilst macrophages activated by immune complexes and TLR ligands *in vitro* express high levels of IL-10 and low levels of Arg1 [62]. In our hands, antibodies together with helminth larvae elicited potent Arg1 expression even in the absence of IL-4Rα signaling. Interestingly, immune complexes were previously shown to induce a robust FcR- and complement-mediated activation of C/EBPbeta, and C/EBPbeta has previously been associated with Arg1 induction in response to bacterial infection [63] [45], indicating it may also play a role in our model. Importantly the prominent expression of Arg1 by helminth-antibody activated macrophages did not correlate with other markers typical of AAM, such as Relmα. Thus the helminth-antibody activated macrophages described in this study seem to represent a novel “regulatory” macrophage type, which expresses Arg1 and wound healing genes in the absence of Relmα. The exact role of these helminth-antibody activated macrophages in tissue repair during the resolution phase of intestinal helminth infection would be of great interest for future studies.

In addition to the “canonical” pathway of Arg1 induction via the IL-4/IL-13/Stat6 pathway [64], Arg1 expression can be elicited via MyD88/IL-6/Stat3 and adenosine dependent mechanisms [65] [66]. Here, we have identified a novel pathway, which leads to the induction of Arg1 in the context of an adaptive type 2 response and which involves antibody-helminth interactions. The existence of multiple pathways for the induction of Arg1 may serve to achieve redundancy in an essential anti-inflammatory effector mechanism functional in many settings of infection, inflammation and wound healing.

In summary, we have demonstrated that antibodies mediate the activation of macrophages resulting in Arg1 expression and immobilization of tissue invasive helminth larvae. Macrophages often form a large component of the inflammatory infiltrate following allergen challenge or helminth infection, and our data indicate that antibodies form a previously unrecognized component of macrophage regulation during type 2 immune responses.

## Materials and Methods

All animal experiments were approved by the office Affaires vétérinaires (1066 Epalinges, Canton Vaud, Sitzerland) with the authorization Number 2238 according to the guidelines set by the service de la consummation et des affaires vétérinaires federal (Canton Vaud, Switzerland).

### Mice

C57BL/6, BALB/c, J_H_^−/−^, FcRγ-chain^−/−^ [67], C3^−/−^ [68], IgE^−/−^ [69] and IL-4Rα^−/−^ [70] were bred and maintained under specific pathogen free conditions at the Ecole Polytechnique Fédérale (EPFL) de Lausanne, Switzerland. FcγRI/II/III/IV^−/−^ mice were bred and maintained at Leiden University, Netherlands and Arg1^fl/fl^Tie2-Cre [45] and Tie2-Cre [47] mice were bred and maintained at New Jersey Medical School, USA. The following primers were used for genotyping of Arg1^fl/fl^Tie2-Cre mice: wildtype Arg1/Arg1^fl/fl^: 5′-TGCGAGTTCATGACTAAGGTT-3′ (forward), 5′-AAAGCTCAGGTGAATCGG-3′(reverse); Tie2-Cre: 5′-CGCATAACCAGTGAAACAGCATTGC-3′ (forward) 5′-CCCTGTGCTCAGACAGAAATGAGA-3′ (reverse).

### Infection, parasitology and collection of immune serum

C57BL/6 mice were infected with 200 *Hp* L3 larvae by oral gavage. Worms were cleared by treatment with two courses of Cobantril (Interdelta - Givisiez, Fribourg, Switzerland) 28 days after primary infection. 14 days later mice were challenge-infected with 200 larvae. BEC (Cayman Chemicals, Ann Arbor, MI) was administered (0.1 ml, 0.2%) once daily from the first day of infection by oral gavage. Mice were sacrificed on day 4 post infection and immune serum was collected from the inferior vena cava. Adult worms were counted under a stereomicroscope on longitudinally opened small intestines of 1° or challenge-infected mice. Tissue dwelling L4 larvae were counted after recovery with a modified Baermann apparatus [16].

### Histology, immunofluorescence stainings and confocal microscopy

Serial paraffin sections were stained with hematoxylin and eosin. Granulomas were identified by light microscopy and serial sections were used for pathological scoring (details see Text S1) or immunofluorescence staining for Arginase-1 and F4/80. Stained tissue sections were imaged with an inverted point scanning confocal microscope (Zeiss LSM 710) with a Plan-Apochromat (63×/1.4 NA or 40×/1.3 NA) objective and pixel intensities were analysed using a custom built CellProfiler pipeline (for details see Text S1).

### Flow cytometry

*In vitro* cultured cells or cells isolated from granuloma were stained with fluorescently labeled monoclonal antibodies (see Text S1) and acquired on a BD LSRII flow cytometer (BD, Franklin Lakes, NJ).

### Luminescent Cell Viability Assay for assessment of larval viability

Larvae were recovered from co-cultures with macrophages with or without immune serum, washed thoroughly with enzyme free cell dissociation solution (Milipore, Billerica, MA) to remove all adherent cells, counted and homogenized in a cell/tissue homogenizer in RPMI1640 by using 0.1 mm Zirconia/Silica beads (BioSpec Products, Inc.). ATP levels in larval homogenates were analysed by CellTiter-Glo Luminescent Cell Viability Assay (Promega, Madison, WI).

### Culture and treatment of murine bone marrow cells and peritoneal macrophages

Peritoneal macrophages were isolated by plating peritoneal wash cells on petri dishes overnight and by removing non-adherent cells. Bone marrow was flushed from the femur and tibia of wildtype or transgenic mice and passed through cell strainers (70 µm). Cells (10^6^/ml) were cultured in M-CSF (L929) supplemented medium (RPMI, 10% FCS, penicillin/streptomycin, β-mercaptoethanol) for 7 days as described previously [71]. On day 7, macrophages were harvested and stimulated for 24 h as indicated with L3 larvae (500 larvae/10^6^ cells),(10 ng/ml) (Peprotech, Rocky Hill, NJ), or immune sera (1∶50 v/v). For differentiation into eosinophils, bone marrow cells were cultured as described [72] in the presence of Flt3 and SCF (Peprotech, Rocky Hill, NJ) for 4 days and in the presence of IL-5 (Peprotech, Rocky Hill, NJ) for subsequent 8 days. For RNA extraction, cells were re-suspended in Trizol and kept at −80°C.

Adherence of macrophages to larvae was determined by manual counting of bright field microscopy pictures taken with an Olympus AX70 microscope (UPLAN 10×/0.3 NA objective).

### Measurement of larval motility

Time-lapse series (60 s: 20 frames, 3 s intervals) of larvae in the small intestine *ex vivo* or *in vitro* were acquired in order to measure their motility with an Olympus AX70 microscope (UPLAN 10×/0.3 NA objective). Larval motility was quantified by using custom-made Fiji macros (for details see Text S1).

### qPCR and microarray analysis

RNA was extracted with a Direct-zol RNA MiniPrep kit (Zymo Research, Irvine, CA) and reverse transcribed using RevertAid cDNA synthesis reagents (Thermo Scientific, Waltham, MA) for qPCR analysis. QPCR was performed using SYBR Green I Master Mix (Eurogentec, Liege, Belgium) on an Applied Biosystems 7900HT System (for qPCR primer sequences see Table S1). Microarray analysis was performed using Affymetrix mouse arrays (Affymetrix, Santa Clara, CA, USA) (for details see Text S1).

### ELISA

Concentrations of IL-4 or IL-13 in cell culture supernatants or mouse serum were quantified by using ELISA Ready-SET-Go! Kits (eBioscience, San Diego, CA).

### Arginase-1 activity assay

Macrophage arginase-1 activity was determined according to previously published methods [73]. Briefly, adherent macrophages were lysed and conversion of L-arginine was quantified indirectly by measuring urea production with a QuantiChrom Urea Assay (BioAssay Systems, Hayward, CA).

## Supporting Information

Figure S1 **Image processing for motility analysis** ***ex vivo*** **and** ***in vitro*** **and quantification of immune-fluorescent staining for Arg1 and F4/80.** (A) First (a) and last (a′) frame of an *ex vivo* time-lapse acquisition. Mask obtained for the first (b) and last (b′) frame based on a manual drawing. XOR operator on first and last frame masks (c). Common parts to both masks become null (black) due to the XOR operation. (B) Montage of time-lapses series of a larva co-cultured with macrophages (Arg1^f/f^Tie2-Cre) and immune serum *in vitro* (10 frames, 3 s interval). (C) Steps of the image processing. (a) Raw images, (b) “find edges” filtered images, (c) “median filter” filtered image, (d) “threshold manually selected” segmented image. (D) Temporal color code of the z-projection of mask series using the “Spectrum” look-up table (a). Z-Projections of mask (b, b′), contour (c, c′) and convex hull (d, d′) obtained for the time-lapses series from an *in vitro* experiment using knock-out (KO) (Arg1^f/f^Tie2-Cre) (b, c, d) or wildtype (WT) (C57BL/6) macrophages (b′, c′, d′). (E) Original DAPI staining image (a), nuclei identified (b) and their outlines on the DAPI staining image (c); expanded nuclei mask used to measure in F4/80 and Arg1 channels (d).
(PDF)

Figure S2 **Except for basophils, granuloma cell populations are largely overlapping in challenge** ***Hp*** **infected wildtype and antibody deficient mice.** (A) Gating strategy for the flow cytometry analysis of granuloma cell populations; (B) Characterisation of granuloma cell populations in C57BL/6 and J_H_^−/−^ mice on day 4 post challenge infection according to the gating strategy in (A). (C) MFI of CD11b, IgG1 and IgG3 on granuloma macrophages from challenge infected C57BL/6 and FcRγ^−/−^ mice. Pooled data from two independent experiments with 4–6 mice per group are shown as mean + SEM (*p<0.05, **p<0.01).
(PDF)

Figure S3 **Immune serum in combination with** ***Hp*** **larvae induces the expression of genes involved in granulocyte recruitment and activation, T_H_2 responses and wound healing and purified 2° IgG in combination with naïve serum upregulates Arg1 expression.** mRNA levels normalized to GAPDH expression and relative to untreated cells for *Cxcl3* (A), *Cxcl2* (B), *Il33* (C), *Jag1* (D), *S100A8* (E), *Emp2* (F), *Tpbpa* (G), *Trem1* (H), *Inhba* (I) or *Arg1* (J). (A–I) Expression of the ten most-upregulated genes in BMMac treated with immune serum and larvae *versus* larvae alone identified by microarray was analysed by qPCR using cDNA from BMMac from C57BL/6 or FcRγ^−/−^ mice cultured in the presence or absence of larvae and/or immune serum. (J) Expression of *Arg1* after treatment with purified 2° IgG −/+ naïve serum. Pooled data from three independent experiments with bone marrow from 2–3 mice per group are shown as mean + SEM (*p<0.05, **p<0.01, ***p<0.001, Mann-Whitney test).
(PDF)

Figure S4 **Antibody-induced adherence to** ***Hp*** **larvae occurs in different types of macrophages but not eosinophils and is independent of IgE but dependent on Fc receptors and IgG.** (A) Adherence of peritoneal macrophages to larvae in response to immune serum from 1° and challenge *Hp* infected C57BL/6 mice. (B) Immune serum does not induce adherence of bone marrow derived eosinophils to *Hp* larvae. (C/D) Eosinophils fail to immobilize larvae. (E) FcγRI/II/III/IV^−/−^ macrophages show reduced larval trapping. (F) Surface levels of IgG, IgM, CD16/32 (FcγRIII/II) and CD64 (FcγRI) on C57BL/6 and FcγRI/II/III/IV^−/−^ macrophages was analysed by flow cytometry; (G/H) Immune serum from IgE^−/−^ mice efficiently induces adherence (G) and immobilization (H); CD16 deficient macrophages show normal immune serum induced adherence (G) and a minor defect in larval immobilization (H); Peritoneal macrophages from C57BL/6 mice or BM-derived macrophages or eosinophils from C57BL/6, BalbC, or FcγRI/II/III/IV^−/−^ mice were co-cultured with larvae in the presence or absence of immune serum from *Hp* infected C57BL/6, BalbC or IgE^−/−^ mice for 24 h. Adherent macrophages per larva were counted in light microscopy images. Larval motility was quantified by Fiji (as described in Experimental Procedures). Immune serum activated BMMac were stained for surface IgG, IgM and antibody receptors. Pooled data and representative histograms from two independent experiments with peritoneal wash or bone marrow from 2–4 mice per group are shown as mean + SEM (*p<0.05, **p<0.01, ***p<0.001, Mann-Whitney test).
(PDF)

Figure S5 **Presence of IL-4 does not change adherence or larval viability in co-cultures with macrophages and immune serum and co-culture supernatants contain negligible levels of IL-4 and IL-13; Genotyping and deletion efficiency for Arg1^f/f^Tie2-Cre mice.** (A-C) BMMac were cultured with larvae or larvae and immune serum (IS) in the presence or absence of IL-4 (10 ng/ml) for 24 h. (A/B) Macrophage adherence and larval motility were determined by light microscopy. (C) Larval viability was assessed by CellTiter-Glo Assay and normalized to the larval viability after culture for 24 h in BMMac medium without BMMac or IS. (D-G) IL-4 or IL-13 in serum (D/E) or cell culture supernatants (F/G) was quantified by ELISA. (H/I) Arg1^f/f^, WT Arg1 or Tie2-Cre transgene expression was determined in tissue biopsies from Arg1^f/f^Tie2-Cre or Tie2-Cre mice. (J) Relative expression of Arg1 mRNA was determined in BMMac from the same mice as in H/I. Data from two independent experiments are shown as mean + SEM (***p<0.001, Mann-Whitney test).
(PDF)

Movie S1 ***In vivo*** **motility of larvae in granuloma of antibody and FcRγ deficient versus wildtype mice.** A piece of the upper duodenum of challenge *Hp* infected mice was removed at day 4 p.i. and carefully flattened between two glass slides for imaging with an Olympus AX70 microscope (10× objective). Movies of granulomas, containing clearly visible larvae were recorded over a time frame of 60 s (20 frames). Movies were processed using iMovie software. Representative examples are shown.
(M4V)

Movie S2 **FcRγ and C3 dependent pathways contribute to immune serum induced trapping of** ***Hp*** **larvae by BMMac.** Larvae were incubated with BMMac from C57BL/6 or FcRγ^−/−^ mice in the presence of immune serum from C57BL/6 or FcRγ*C3^−/−^ mice for 24 h. Suspensions from co-cultures were transferred to glass slides, carefully covered with cover slips and time lapse-movies were immediately recorded with an Olympus AX70 microscope. Movies were processed using iMovie software. Representative examples are shown.
(M4V)

Movie S3 **Immune serum promotes adherence of BMMac to L4 larvae** ***in vitro*** **.** Larvae recovered from the small intestine of challenge-infected mice were incubated with BMMac in the presence of or absence of immune serum for 24 h. Suspensions from co-cultures were transferred to glass slides, carefully covered with cover slips and time lapse-movies were immediately recorded with an Olympus AX70 microscope. Movies were processed using iMovie software. Representative examples are shown.
(M4V)

Movie S4 **Arg1 deficiency impairs immune serum induced trapping of larvae by BMMac** ***in vitro*** **.** Larvae were incubated with BMMac from Arg1^f/f^Tie2-Cre or Tie2-Cre mice in the presence of immune serum from challenge *Hp* infected C57BL/6 mice for 24 h. Suspensions from co-cultures were transferred to glass slides, carefully covered with cover slips and time lapse-movies were immediately recorded with an Olympus AX70 microscope. Movies were processed using iMovie software. Representative examples are shown.
(M4V)

Movie S5 **The arginase inhibitor BEC prevents larval immobilization during challenge infection** ***in vivo*** **.** C57BL/6 mice were treated with S-(2-boronoethyl)-L-cysteine (BEC) (0.2%, 100 µl by oral gavage) during challenge *Hp* infection. Mice were sacrificed on day 4 p.i. and small intestines were removed. Pieces of the upper duodenum were carefully covered with cover slips and time lapse-movies were immediately recorded with an Olympus AX70 microscope. Movies were processed using iMovie software. Representative examples are shown.
(M4V)

Movie S6 **L-ornithine and polyamines reduce larval motility** ***in vitro*** **.** Larvae were incubated in BMMac medium supplemented with L-ornithine or polyamines (100 µM) for 24 h, aliquots were transferred to glass slides and time lapse movies were recorded immediately with an Olympus AX70 microscope. Movies were processed using iMovie software. Representative examples are shown.
(M4V)

Text S1 **Supporting Materials and Methods and qPCR primer sequences.** Detailed descriptions of experimental procedures, materials and data analysis methods including Fiji macros and Cell profiler pipelines can be found in the Supporting Information (Text S1).
(DOCX)
