# Supplementary figures and images for: Antibodies Trap Tissue Migrating Helminth Larvae and Prevent Tissue Damage by Driving IL-4Rα-Independent Alternative Differentiation of Macrophages
Source: PLoS Pathog. 2013 Nov 14;9(11):e1003771. doi: 10.1371/journal.ppat.1003771 (PMC3828184; doi:10.1371/journal.ppat.1003771)

Fig.S1

A

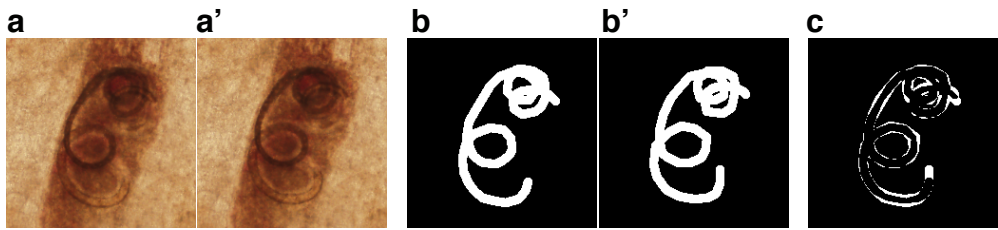

B

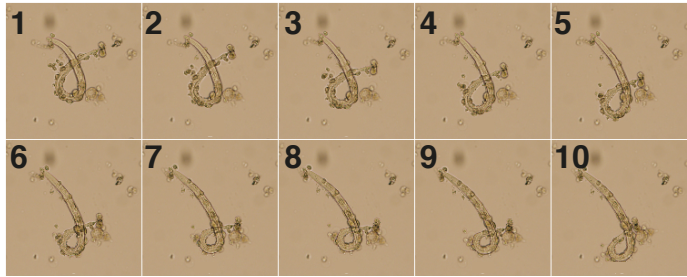

C

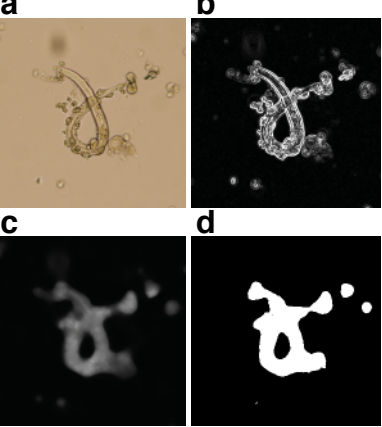

D

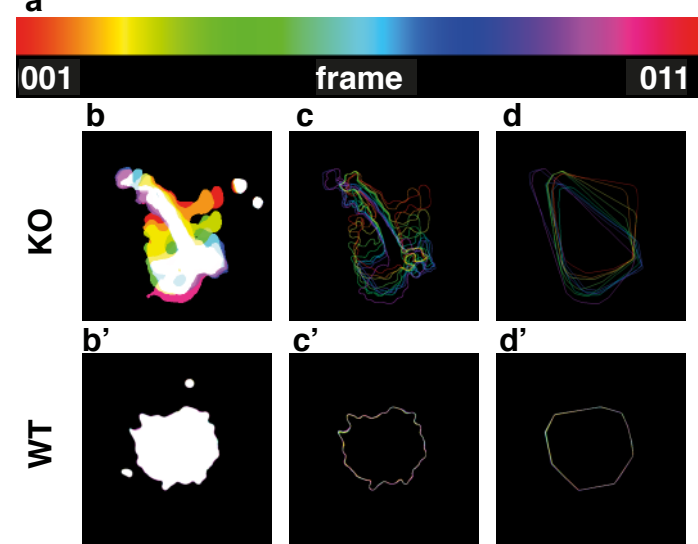

E

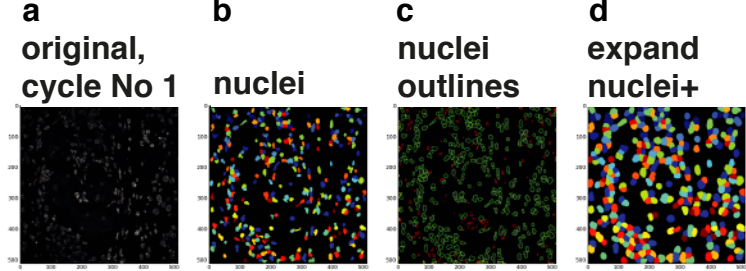

Supplement: Figure S1 — Image processing for motility analysis ex vivo and in vitro and quantification of immune-fluorescent staining for Arg1 and F4/80. (A) First (a) and last (a′) frame of an ex vivo time-lapse acquisition. Mask obtained for the first (b) and last (b′) frame based on a manual drawing. XOR operator on first and last frame masks (c). Common parts to both masks become null (black) due to the XOR operation. (B) Montage of time-lapses series of a larva co-cultured with macrophages (Arg1f/fTie2-Cre) and immune serum in vitro (10 frames, 3 s interval). (C) Steps of the image processing. (a) Raw images, (b) “find edges” filtered images, (c) “median filter” filtered image, (d) “threshold manually selected” segmented image. (D) Temporal color code of the z-projection of mask series using the “Spectrum” look-up table (a). Z-Projections of mask (b, b′), contour (c, c′) and convex hull (d, d′) obtained for the time-lapses series from an in vitro experiment using knock-out (KO) (Arg1f/fTie2-Cre) (b, c, d) or wildtype (WT) (C57BL/6) macrophages (b′, c′, d′). (E) Original DAPI staining image (a), nuclei identified (b) and their outlines on the DAPI staining image (c); expanded nuclei mask used to measure in F4/80 and Arg1 channels (d). (PDF) [file ppat.1003771.s001.pdf]

**Fig.S2****A**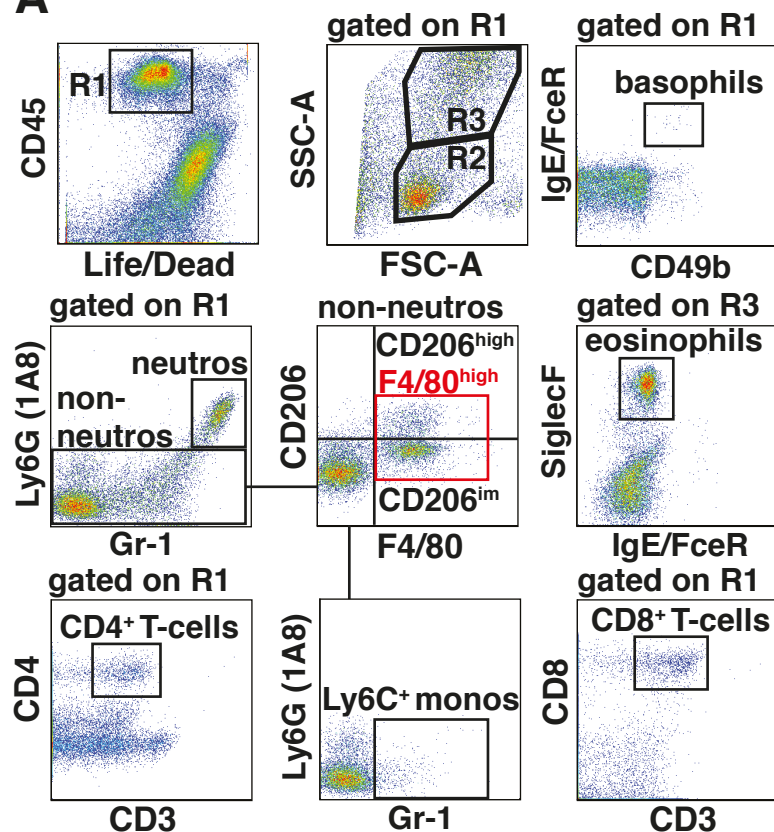**C**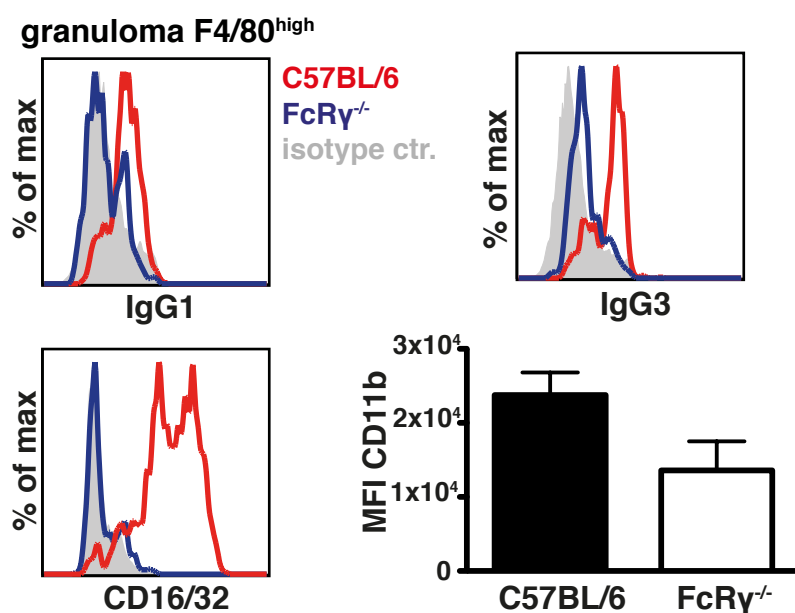**B**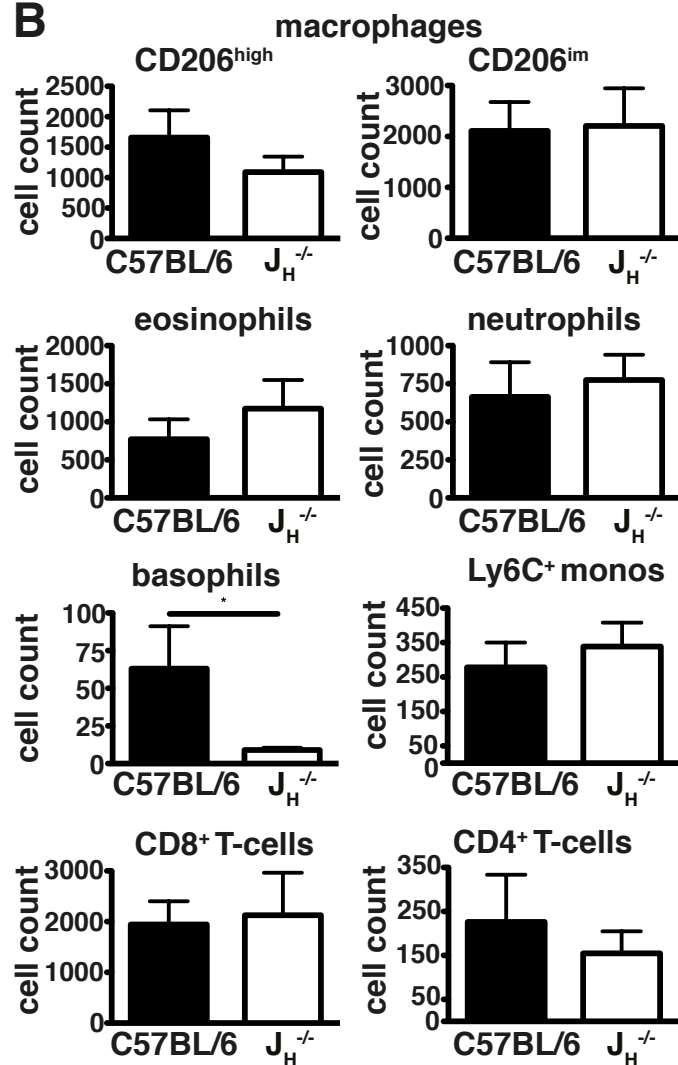

Supplement: Figure S2 — Except for basophils, granuloma cell populations are largely overlapping in challenge Hp infected wildtype and antibody deficient mice. (A) Gating strategy for the flow cytometry analysis of granuloma cell populations; (B) Characterisation of granuloma cell populations in C57BL/6 and JH −/− mice on day 4 post challenge infection according to the gating strategy in (A). (C) MFI of CD11b, IgG1 and IgG3 on granuloma macrophages from challenge infected C57BL/6 and FcRγ−/− mice. Pooled data from two independent experiments with 4–6 mice per group are shown as mean + SEM (*p<0.05, **p<0.01). (PDF) [file ppat.1003771.s002.pdf]

Fig.S3

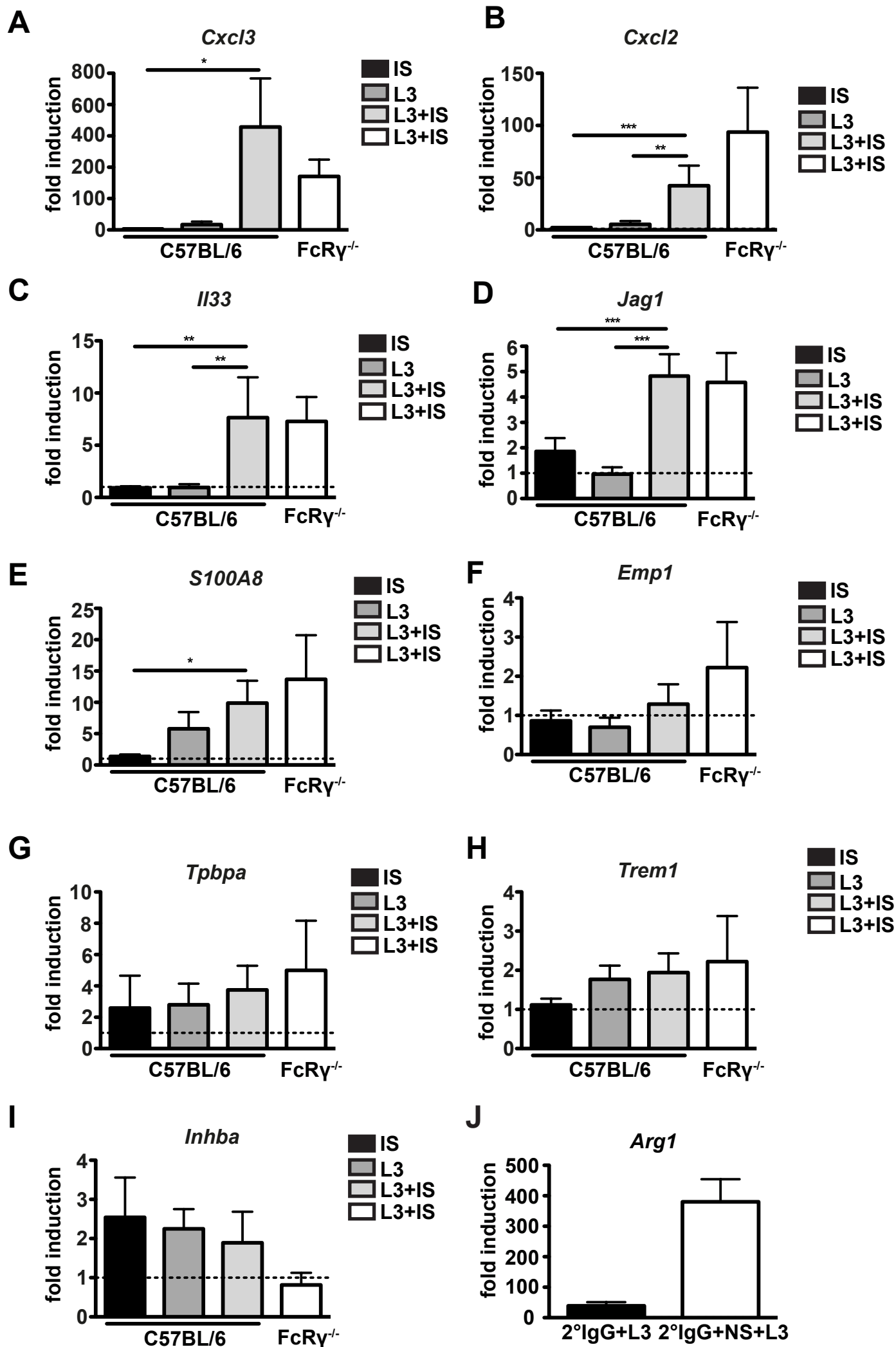

Supplement: Figure S3 — Immune serum in combination with Hp larvae induces the expression of genes involved in granulocyte recruitment and activation, TH2 responses and wound healing and purified 2° IgG in combination with naïve serum upregulates Arg1 expression. mRNA levels normalized to GAPDH expression and relative to untreated cells for Cxcl3 (A), Cxcl2 (B), Il33 (C), Jag1 (D), S100A8 (E), Emp2 (F), Tpbpa (G), Trem1 (H), Inhba (I) or Arg1 (J). (A–I) Expression of the ten most-upregulated genes in BMMac treated with immune serum and larvae versus larvae alone identified by microarray was analysed by qPCR using cDNA from BMMac from C57BL/6 or FcRγ−/− mice cultured in the presence or absence of larvae and/or immune serum. (J) Expression of Arg1 after treatment with purified 2° IgG −/+ naïve serum. Pooled data from three independent experiments with bone marrow from 2–3 mice per group are shown as mean + SEM (*p<0.05, **p<0.01, ***p<0.001, Mann-Whitney test). (PDF) [file ppat.1003771.s003.pdf]

**Fig.S4**

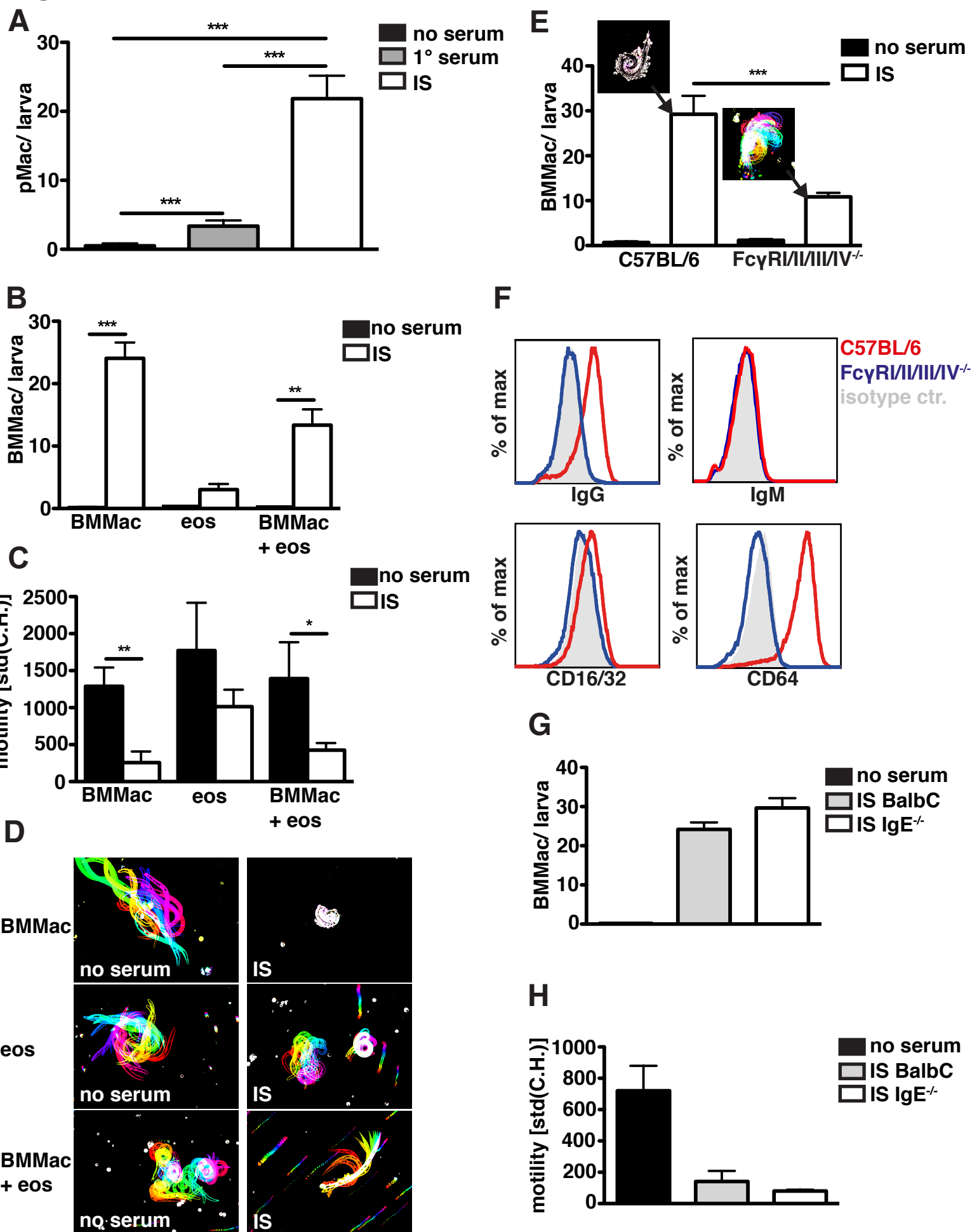

Supplement: Figure S4 — Antibody-induced adherence to Hp larvae occurs in different types of macrophages but not eosinophils and is independent of IgE but dependent on Fc receptors and IgG. (A) Adherence of peritoneal macrophages to larvae in response to immune serum from 1° and challenge Hp infected C57BL/6 mice. (B) Immune serum does not induce adherence of bone marrow derived eosinophils to Hp larvae. (C/D) Eosinophils fail to immobilize larvae. (E) FcγRI/II/III/IV−/− macrophages show reduced larval trapping. (F) Surface levels of IgG, IgM, CD16/32 (FcγRIII/II) and CD64 (FcγRI) on C57BL/6 and FcγRI/II/III/IV−/− macrophages was analysed by flow cytometry; (G/H) Immune serum from IgE−/− mice efficiently induces adherence (G) and immobilization (H); CD16 deficient macrophages show normal immune serum induced adherence (G) and a minor defect in larval immobilization (H); Peritoneal macrophages from C57BL/6 mice or BM-derived macrophages or eosinophils from C57BL/6, BalbC, or FcγRI/II/III/IV−/− mice were co-cultured with larvae in the presence or absence of immune serum from Hp infected C57BL/6, BalbC or IgE−/− mice for 24 h. Adherent macrophages per larva were counted in light microscopy images. Larval motility was quantified by Fiji (as described in Experimental Procedures). Immune serum activated BMMac were stained for surface IgG, IgM and antibody receptors. Pooled data and representative histograms from two independent experiments with peritoneal wash or bone marrow from 2–4 mice per group are shown as mean + SEM (*p<0.05, **p<0.01, ***p<0.001, Mann-Whitney test). (PDF) [file ppat.1003771.s004.pdf]

**Fig.S5**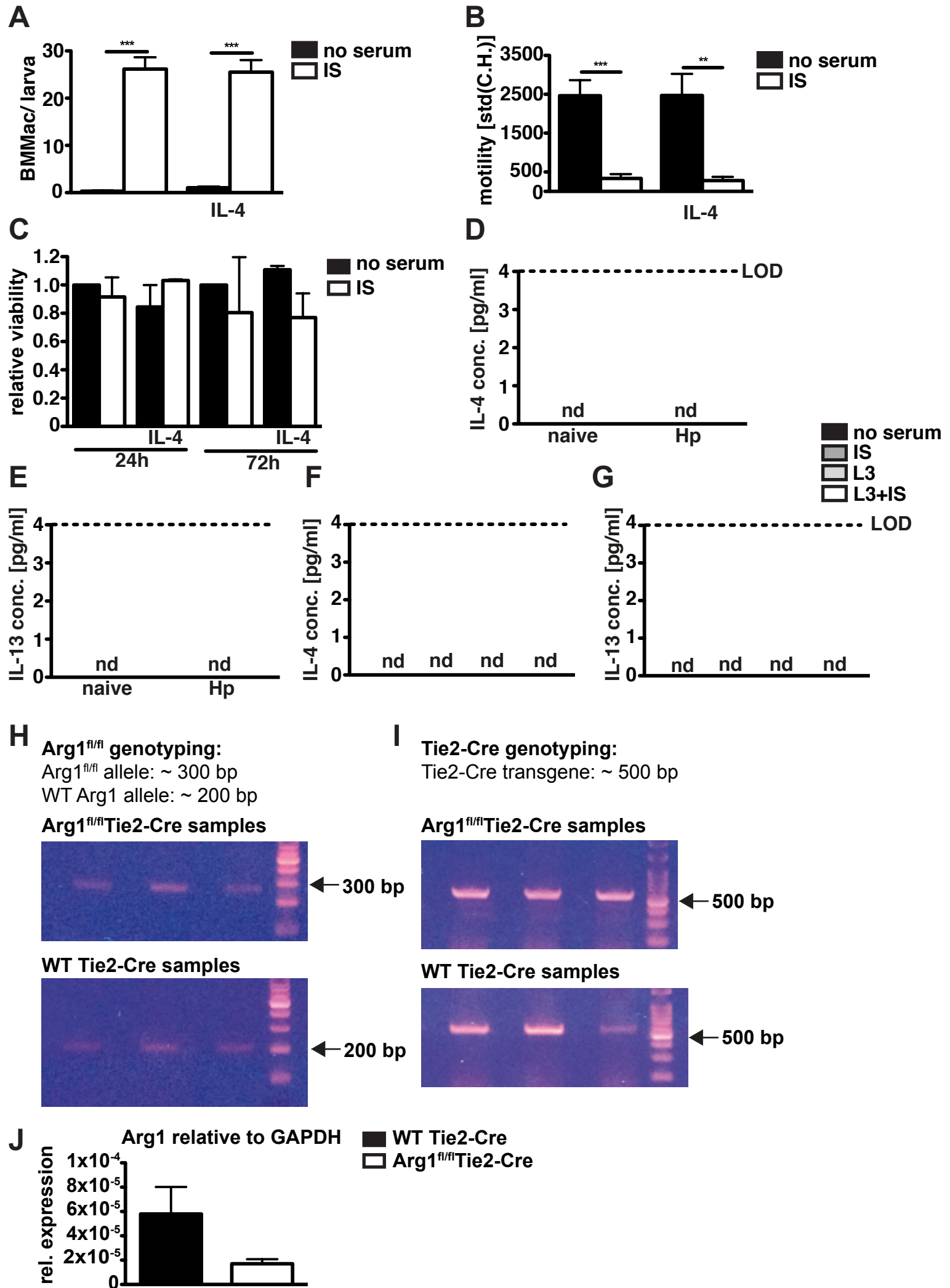

Supplement: Figure S5 — Presence of IL-4 does not change adherence or larval viability in co-cultures with macrophages and immune serum and co-culture supernatants contain negligible levels of IL-4 and IL-13; Genotyping and deletion efficiency for Arg1f/fTie2-Cre mice. (A-C) BMMac were cultured with larvae or larvae and immune serum (IS) in the presence or absence of IL-4 (10 ng/ml) for 24 h. (A/B) Macrophage adherence and larval motility were determined by light microscopy. (C) Larval viability was assessed by CellTiter-Glo Assay and normalized to the larval viability after culture for 24 h in BMMac medium without BMMac or IS. (D-G) IL-4 or IL-13 in serum (D/E) or cell culture supernatants (F/G) was quantified by ELISA. (H/I) Arg1f/f, WT Arg1 or Tie2-Cre transgene expression was determined in tissue biopsies from Arg1f/fTie2-Cre or Tie2-Cre mice. (J) Relative expression of Arg1 mRNA was determined in BMMac from the same mice as in H/I. Data from two independent experiments are shown as mean + SEM (***p<0.001, Mann-Whitney test). (PDF) [file ppat.1003771.s005.pdf]
