# Supplementary material for: Antibodies Trap Tissue Migrating Helminth Larvae and Prevent Tissue Damage by Driving IL-4Rα-Independent Alternative Differentiation of Macrophages
Source: PLoS Pathog. 2013 Nov 14;9(11):e1003771. doi: 10.1371/journal.ppat.1003771 (PMC3828184; doi:10.1371/journal.ppat.1003771)
Supplement: Text S1 — Supporting Materials and Methods and qPCR primer sequences. Detailed descriptions of experimental procedures, materials and data analysis methods including Fiji macros and Cell profiler pipelines can be found in the Supporting Information (Text S1). (DOCX) [file ppat.1003771.s012.docx]

**Text S1: Supporting Information**

**Supporting Materials and Methods**

**Pathological scoring of tissue sections**

Histological sections were scored for degrees of pathology in a blind manner by a board certified veterinary pathologist. Necrosis within granulomas was quantified as percentage (%) on the total area of granuloma, as follows: up to 25% = 1, up to 50% = 2, more than 50% = 3.

**Assessment of larval** **motility *ex vivo* and *in vitro***

The motility *ex vivo* was represented by subtle changes in the larval position within the tissue (difference between the area covered by the larva at t=0 and t=60), whilst the motility *in vitro* was represented by large differences in the larval shape between each frame (e.g. by bending or curling). Thus, the following 2 methods were developed to assess *ex vivo* or *in vivo* motility:

*Ex vivo* microscopy that was performed directly on explanted small intestines delivered an image quality, which was not suitable for an automatic segmentation (low contrast). The semi-automatic approach consists of drawing a line defining the worm, on the first and the last frame. The lines were then expanded in order to create masks. The difference between the two masks (“Exclusive OR operator”; XOR) was calculated. The area remaining after the XOR operation was used as a measure of the larval movement.

*In vitro* time-lapse experiments were semi-automatically processed as followed: raw images were processed with a Sobel edge detector (Fiji, “find edges” filter) to highlight sharp changes in intensity resulting in the worm contours. This was followed by a “median filter” (of radius 10 pixels) in order to smooth the image and facilitate the subsequent thresholding step. The threshold was manually selected (by visually fitting the worms’ edges as best as possible) and the detected particles were filtered (area > 4000 pixel^2^). Based on the masks the “convex hull” – a measure of the larval shape - was computed. Finally, the area-difference between two consecutive frames was used as a measure of the worms’ movement.

**Antibodies used for flow cytometry and immunofluorescence**

The following monoclonal antibodies were used for surface staining for flow cytometric analysis: anti-CD45 PE-Cy5 or Alexa Fluor 700 anti-F4/80 APC, anti-CD206 FITC or biotin, Streptavidin PE-Cy7, anti-CD4 Pacific Blue, anti-CD11b Pacific Blue, anti-CD8 Pe-Cy7, anti-IgE FITC, anti-CD64 PE, anti-CD16/32 PercP-Cy5.5, anti-CD49b Alexa Fluor 647, anti-IgG3 Alexa Fluor 488 (all from BioLegend, San Diego, CA), anti-FceR PE, anti-Siglec F PE, anti-IgG1 FITC (all from BD Biosciences, Franklin Lakes, NJ). Cells from granuloma were stained with Live Dead stain Aqua (Lifetechnologies, Zug, Switzerland) before proceeding to surface stain.

The following primary and secondary antibodies were used for immunofluorescence staining: polyclonal rabbit anti-liver Arginase-1 (Abcam, Cambridge, UK), rat anti-mouse F4/80 (Serotec), Alexa Fluor 488 conjugated donkey anti-rabbit IgG and Alexa Fluor 568 conjugated goat anti-rat (Lifetechnologies, Zug, Switzerland). All tissues were counterstained with DAPI (Lifetechnologies, Zug, Switzerland).

**Cell profiler pipeline for quantification of immunofluorescence staining**

A mask based on the nuclear DAPI staining was created and used to measure the mean pixel intensities of the Arg1 and the F4/80 staining in the respective channels for each identified object. Major details of the pipeline were i) a correction of the illumination calculated on the DAPI staining image (Calculation method: Regular / Dilation Radius : 30 / rescale the illumination function : Yes / Calculated for Each image / No smooting method ), ii) correction of the DAPI image, iii) detection of nuclei using the function “Identify primary objects” (typical diameter: 10-30 / discard objects outside the range and touching the border / Thresholding method : Otsu Global , 3 classes, weighted variance, Background / No threshold correction / Laplacian of Gaussian to distinguish clumped objects) iv) expansion of nuclei by 5 pixels, and v) measurement of the pixel intensities for other channels were performed using this enlarged nuclei mask. The entire CellProfiler pipeline can be downloaded at *https://documents.epfl.ch/users/e/es/esser/www/macros_Esseretal/*.

**Microarray analysis**

Prior to microarray analysis, all RNA quantities were assessed by using a NanoDrop ND-1000 spectrophotometer and the RNA quality was assessed using RNA 6000 NanoChips with the Agilent 2100 Bioanalyzer (Agilent, Palo Alto, USA). For each sample, 300ng of total RNA were amplified using the message amp II enhanced (AM1791, ambion) kit. 12.5μg of the resulting biotin-labelled cRNA was chemically fragmented. Affymetrix mouse arrays (Affymetrix, Santa Clara , CA, USA) were hybridized with 11μg of fragmented target, at 45°C for 17 hours washed and stained according to the protocol described in Affymetrix GeneChip Expression Analysis Manual (Fluidics protocol FS450_0007).

The arrays were scanned using the GeneChip Scanner 3000 7G (Affymetrix) and raw data was extracted from the scanned images and analyzed with the Affymetrix Power Tools software package (Affymetrix).

All statistical analysis were performed using the free high-level interpreted statistical language R and various Bioconductor packages (http://www.Bioconductor.org). Hybridization quality was assessed using the Expression Console software (Affymetrix). Normalized expression signals were calculated from Affymetrix CEL files using RMA normalization method. Differential hybridized features were identified using Bioconductor package “limma” that implements linear models for microarray data [1]. A list of genes that were identified to be differentially expressed between macrophages that were cultured with larvae alone or with a combination of larvae and immune serum can be downloaded at:

https://dm.genomespace.org/datamanager/file/Home/jesser/microarray_macs_L3IS_esser.xlsx

Username: jesser

Password: arginase1

**Table S1: qPCR primer sequences**

| **Gene name** | **Sequence forward primer 5’-3’** | **Sequence reverse primer 5’-3’** |
| --- | --- | --- |
| *Arg1* | GCAACCTGTGTCCTTTCTCC | TCTACGTCTCGCAAGCCAAT |
| *Gapdh* | GGGTGTGAACCACGAGAAAT | CCTTCCACAATGCCAAAGTT |
| *Jag1* | GAGGCGTCCTCTGAAAAACA | ACCCAAGCCACTGTTAAGACA |
| *Il33* | CAATCAGGCGACGGTGTGGATGG | TCCGGAGGCGAGACGTCACC |
| *Cxcl3* | CCATCCAGAGCTTGACGGTGAC | GGCTCAGCTGGACTTGCCGCTC |
| *Cxcl2* | TCAAGGGCGGTCAAAAAGTT | TCCTCCTTTCCAGGTCAGTTA |
| *S100a8* | CACCATGCCCCCTCTACAAGAATGA | CTCTGCTACTCCTTGTGGCTGT |
| *Emp2* | TCCTCTCCACCATTCTCT | AAACCTCTCTCCCTGCTTCA |
| *Tpbpa* | CAGGTACTTGAGACATGACTC | GGCAGAGATTTCTTAGACAATG |
| *Trem1* | GAGCTTGAAGGATGAGGAAGGC | CAGAGTCTGTCACTTGAAGGTCAGTC |
| *Inhba* | TGAATGAACTCATGGAGCAGACC | AGCTGGCTGGTCCTCACAG |

**References**

1. Smyth GK (2004) Linear models and empirical bayes methods for assessing differential expression in microarray experiments. Stat Appl Genet Mol Biol 3: Article3. doi:10.2202/1544-6115.1027.
